# Proteome quantification of cotton xylem sap suggests the mechanisms of potassium-deficiency-induced changes in plant resistance to environmental stresses

**DOI:** 10.1038/srep21060

**Published:** 2016-02-16

**Authors:** Zhiyong Zhang, Maoni Chao, Sufang Wang, Jingjing Bu, Juxiang Tang, Fei Li, Qinglian Wang, Baohong Zhang

**Affiliations:** 1 Henan Collaborative Innovation Center of Modern Biological Breeding, School of Life Science and Technology, Henan Institute of Science and Technology, Xinxiang, 453003, China; 2 Department of Biology, East Carolina University, Greenville, NC27858, US

## Abstract

Proteomics was employed to investigate the molecular mechanisms of apoplastic response to potassium(K)-deficiency in cotton. Low K (LK) treatment significantly decreased the K and protein contents of xylem sap. Totally, 258 peptides were qualitatively identified in the xylem sap of cotton seedlings, of which, 90.31% were secreted proteins. Compared to the normal K (NK), LK significantly decreased the expression of most environmental-stress-related proteins and resulted in a lack of protein isoforms in the characterized proteins. For example, the contents of 21 Class Ш peroxidase isoforms under the LK were 6 to 44% of those under the NK and 11 its isoforms were lacking under the LK treatment; the contents of 3 chitinase isoforms under LK were 11–27% of those under the NK and 2 its isoforms were absent under LK. In addition, stress signaling and recognizing proteins were significantly down-regulated or disappeared under the LK. In contrast, the LK resulted in at least 2-fold increases of only one peroxidase, one protease inhibitor, one non-specific lipid-transfer protein and histone H_4_ and in the appearance of H_2_A. Therefore, K deficiency decreased plant tolerance to environmental stresses, probably due to the significant and pronounced decrease or disappearance of a myriad of stress-related proteins.

Potassium is a macronutrient that participates in many physiological processes, such as osmotic adjustment, photosynthesis, transport and enzyme activation in plants^1^. Potassium deficiency can directly lower various crop plant productivities and qualities^2^,^3^, which may be indirectly reduced via a combination of biotic and abiotic stresses.

In general, a high K status in crops decreases the incidence of diseases and pests^4^,^5^,^6^,^7^. For example, in K-deficient soils, cotton and other crops can be susceptible to *Fusarium* wilt and root rot caused by *Fusarium oxysporum* sp. The application of K either before or after planting is equally effective in reducing this incidence^5^. In rice, increased K supply results in increased resistance to brown leaf spot disease and bacterial leaf blight^8^. Similarly, higher K supply successfully suppresses disease incidence in soybean and wheat^9^,^10^.

Improving the K nutritional status of plants may be very important for the survival of crop plants under abiotic stress conditions, such as drought, chilling, salt stress and high light intensity^11^,^12^. For example, frost damage is inversely related to the available K content in soils and the K concentration in potato leaves; potassium fertilization increases frost resistance in the three K-availability soils, particularly for the soil with the lowest K status^13^. Similar effects were reported by Sharma and Sud^14^. Hakerlerler *et al.*^15^ observed that increasing the amount of K fertilizer increases low-temperature stress tolerance, resulting in as much as 2-fold increases in the yield for various non-greenhouse-grown vegetable crops (tomato, pepper, and eggplant) at temperatures of from 4–16 °C.

The apoplast, which includes the xylem sap, has received specific attention because it constitutes the first barrier to biotic and abiotic stresses. This occurs through defense, recognition, and signaling information to cells for further response^16^. Several research groups have characterized the processes related to plant defense to biotic stresses^17^,^18^,^19^,^20^ and abiotic stresses^21^,^22^,^23^ by analyzing the apoplastic protein fractions.

In general, potassium deficiency can decrease both biotic and abiotic resistance abilities, though there is currently no analysis of the proteome in the apoplast to corroborate these decreases. Understanding how these decreases occurs and the corresponding mechanisms involved are important for preventing problems associated with lowered resistance. This understanding also provides a good reference point for increasing plant resistance to biotic and abiotic stresses under potassium-deficient conditions. Previous work has demonstrated that many proteins related to environmental stress are found in cotton xylem sap^24^. The aim of this study is to qualitatively and quantitatively analyze changes in xylem sap proteins, especially proteins that are related to biotic and abiotic stresses under potassium-deficient conditions, and to further investigate the mechanisms controlling the decreased potassium-deficiency-induced defense ability.

## Results

### Potassium-deficient treatment affected mineral contents, physiological traits and cotton seedling growth

When cotton seedlings were first cultivated under normal K levels for 3 d, subsequent potassium deficiency for 7 d significantly decreased the K content in the root, old leaf (cotyledon) and new leaf (forth true leaf) components, although substantial increases in the micronutrient (Ca, Mg, Fe, Cu, and Zn) contents in corresponding organs were found; however, there was a non-significant change in Cu and Zn in the new leaf component. It addition, this cultivation significantly decreased the K concentration and significantly increased the Ca, Mg and Zn concentrations in the xylem sap (Table 1).

**Figure 1:**
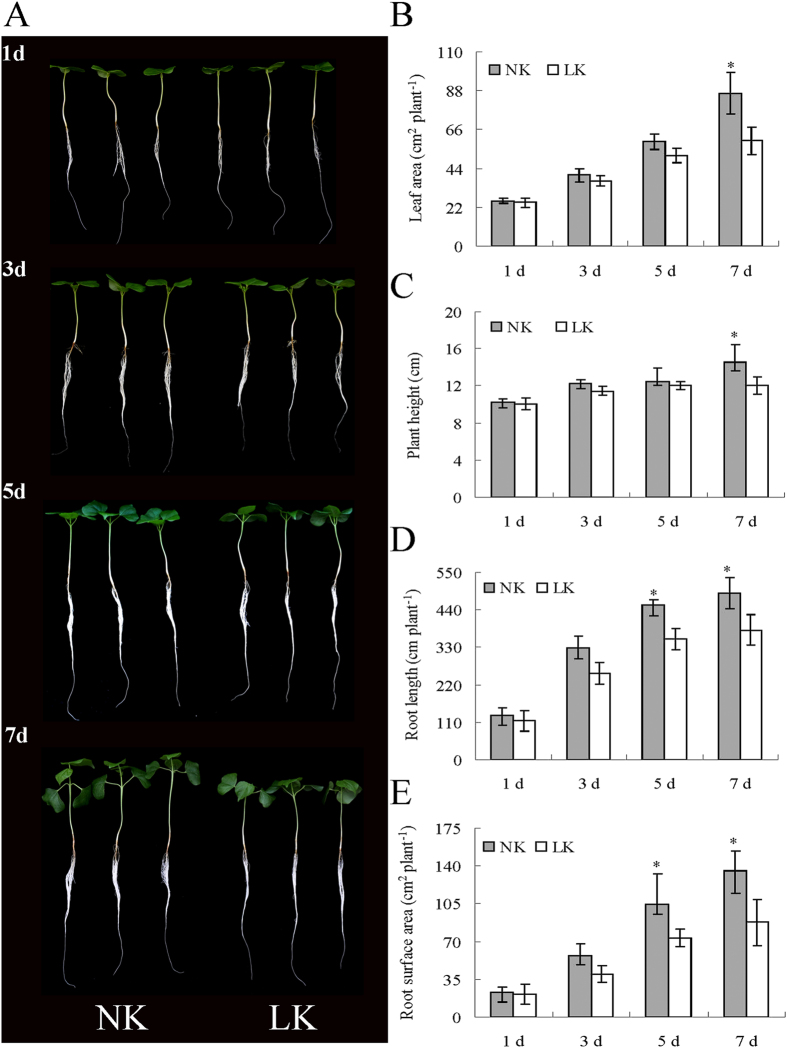
Photograph of comparative growth performances and morphological data of cotton seedlings after different days of LK treatments along with control. Emerging cotton seedlings in wet sand were transferred into a normal solution, grown for 3 d, and separated into a K-deficient solution and a new normal solution and grown for 7 d. Left (**A**): photograph of comparative growth performances of cotton seedlings after 1, 3, 5, 7 d of LK treatments along with control; Right: morphological data of cotton seedlings after 1, 3, 5, 7 d of LK treatments along with control (**B**): Leaf area; (**C**): Plant height; (**D**): Root length; (**E**): Root surface area.

The potassium deficiency of the plant significantly decreased the soluble protein content, the activities of guaiacol-peroxidase (GPX; Class Ш peroxidase; EC1.11.1.7) and superoxide dismutase (SOD; EC1.15.1.1), and pH in the xylem sap, although a significant increase in the xylem sap volume, probably resulting from high root pressure induced by the decreased K content, was also found (Table 3, [Table t2], [Table t3] and supplementary Fig. S2). Under prolonged K deficiency, the growth of cotton seedlings was gradually inhibited. At 5 d of K deficiency, the root length and surface area began to significantly decrease compared to potassium-sufficient plants. At 7 d, the potassium-deficient plants had significantly reduced leaf area, plant height, dry, leaf, stem, root weights, compared to the potassium-sufficient plants (Table 2; Fig. 1).

### General situation of qualitative and quantitative proteins in the cotton xylem sap

In total, 258 qualitative peptides were identified in the xylem sap of the cotton seedlings, including 72 uncharacterized proteins. Among the qualitative peptides, 116 catered to the conditions of quantitative peptides based on a comparison between the low K (LK) and normal K (NK) treatments; 41 and 5 proteins were not detectable in the LK and NK treatments, respectively (Table 4).

**Figure 2:**
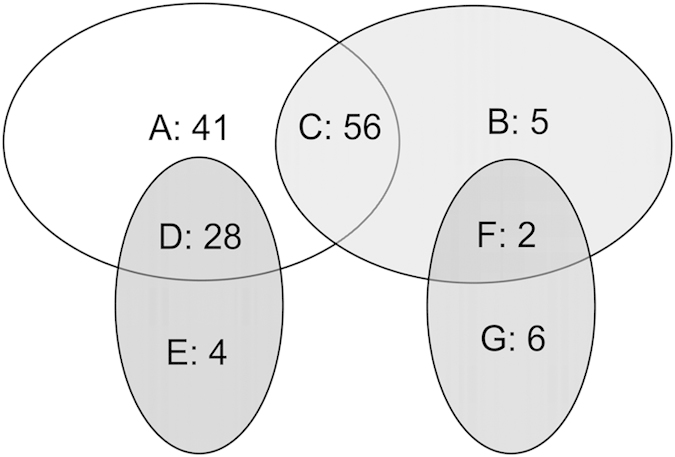
Xylem sap proteins that were qualified but did not meet the comparative quantification requirements between the normal K and low K treatments. Emerging cotton seedlings in wet sand were transferred into a normal solution, grown for 3 d, and separated into a K-deficient solution and a new normal solution and grown for 7 d. These cotton seedlings were used for xylem sap sampling, and the identified proteins not meeting the comparative quantification requirements were statistically classified.

Proteins that had ≥2 detection signals in three NK replicates and no detection signal in three LK replicates were defined as being non-detectable in LK (NLK) (A). Proteins with ≥2 detection signals in three LK replicates and no detection signal in three NK replicates were defined as being non-detectable in NK (NNK) (B). There were 56 proteins with no detection signal in three LK replicates and no detection signal in three NK replicates (C). These proteins may have been caused by no concentration or a low concentration of quantitative peptides. Moreover, there were 28 proteins with only one detection signal in three NK replicates and no detection signal in three LK replicates (D). There were 4 proteins with only one detection signal in three NK replicates and ≥1 detection signals in three LK replicates (E). There were 2 proteins with only one detection signal in three LK replicates and no detection signal in three NK replicates (F). Lastly, there were 6 proteins with only one detection signal in three LK replicates and ≥1 detection signals in three NK replicates (G) (Fig. 2).

### Identification of classical and non-classical secreted proteins in cotton xylem sap

The proportion of secreted proteins within the total was 90.31%; for classical peptides (predicted with a signal peptide by SignalP), the ratio was 56.98%. For the quantitative peptides in comparison, the rate was 93.97%, and the ratio for classical peptides was 68.97% (Table 5). The proteins that were not predicted as being secreted in the latter category (predicted as another type according to TargetP) included 5 uncharacterized proteins and secreted protein isoforms, such as “O-glycosyl hydrolases family 17 protein isoform 1” and “glycosyl hydrolase superfamily protein isoform 3”; two protein fragments were found, i.e., “peroxidase 6 (Fragment)” and “chaperonin CPN60-like protein (Fragment)”; lactoylglutathione lyase; and histone 4 (Table 6, Supplemental Table 1) were also found.

### Protein classification and their regulation by potassium deficiency in the xylem sap

For the quantitative or lacking proteins in the LK or NK treatments, identified proteins were classified as pathogenesis-related (PR) proteins, oxido-reduction-related proteins, signaling proteins, other stress-related proteins, cell wall metabolism proteins, proteins with interacting domains, miscellaneous proteins and uncharacterized proteins. PR proteins were dominant, including antifungal protein (PR-1), β -1, 3-glucanases (PR-2) , chitinases (PR-3 (-4, 8, and 11), thaumatin-like proteins (PR-5), protease inhibitors (PR-6), endo-proteases (PR-7), peroxidases (PR-9), and non-specific lipid transfer proteins (PR-14). Other stress-related proteins included heparanase (putative), chaperonin CPN60-like protein (fragment), lactoylglutathione lyase, and histones. Heparanase was previously found in apoplastic fluid (AF) of poplar, maize and grapevine^16^ and is indirectly involved in H_2_O_2_ degradation. In addition, heparanase generates phenolic compounds that may be used for cell wall fortification. Histones, such as H_2_A and H_4_, are components of the extracellular defense system in plant root tips^25^,^26^. Lactoylglutathione lyase, which is also known as glyoxalase I, is involved in the detoxification of methylglyoxal and the other reactive aldehydes that are produced as a normal component of metabolism and positively responds to salinity, drought and coldness stresses^27^.

Potassium deficiency resulted in 30 significantly decreasing PR proteins, 15 lacking proteins and 8 significantly up-regulated proteins among the 79 sampled PR proteins. Among the sub-classified PR proteins, the sum of significant down-regulation and NLK was substantially higher than that of significant up-regulation and NNK, especially for PR-9 (although this was not the case for PR-14). An analysis of peroxidases in the cotton xylem sap showed that potassium deficiency resulted in 15 proteins exhibiting a significant decrease, 11 that were not detected, 11 with no significant change and only 2 with significant up-regulation. For PR-14, potassium deficiency resulted in 4 proteins exhibiting a significant increase and 1 with no significant change. Although one PR-5 protein was significantly up-regulated and another was significantly down-regulated, the up-regulation margin was much lower than the down-regulation margin (Tables 6 and 7; Supplemental Table 1).

### Evolutionary relationship of peroxidases (EC1.1.11.7) in the xylem sap

**Figure 3:**
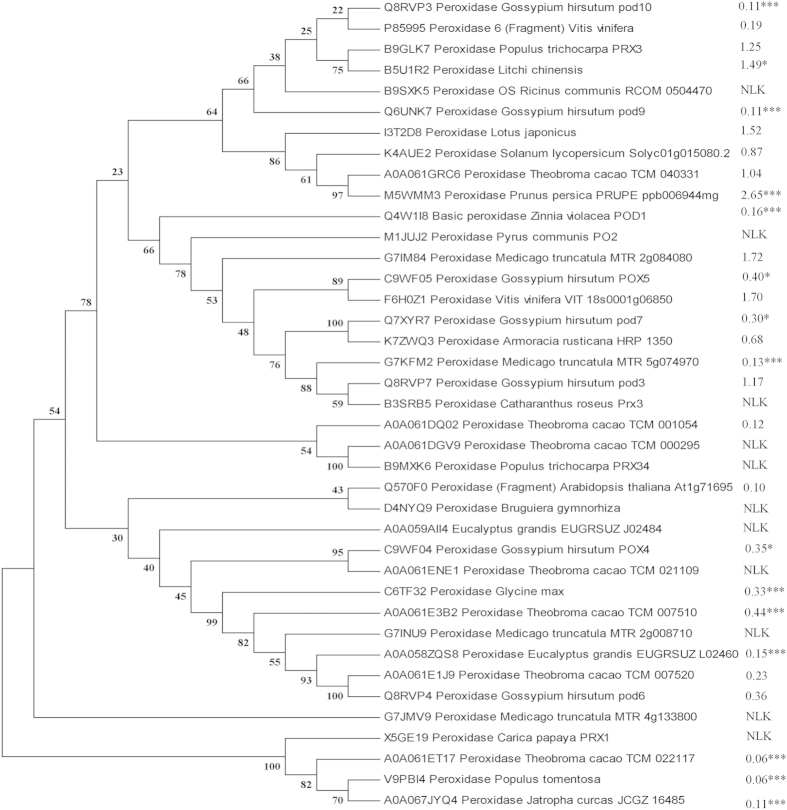
Evolutionary relationships of peroxidases (EC1.1.11.7) in the xylem sap. Emerging cotton seedlings in wet sand were transferred into a normal solution, grown for 3 d, and separated into a K-deficient solution and a new normal solution and grown for 7 d. These cotton seedlings were used for xylem sap sampling, and all peroxidase isoforms were involved in the evolutionary analysis.

Thirty-nine peroxidases in the xylem sap were identified and analyzed according to their evolutionary relationship. Phylogenetic analysis showed that the significantly decreased and non-detectable peroxidases were phylogenetically closer to each other, although the proteins that were not significantly changed were located in the same clades with some of the peroxidases (Fig. 3).

### Coordination of protein (listed in Table 6) expression and corresponding gene expression

**Figure 4:**
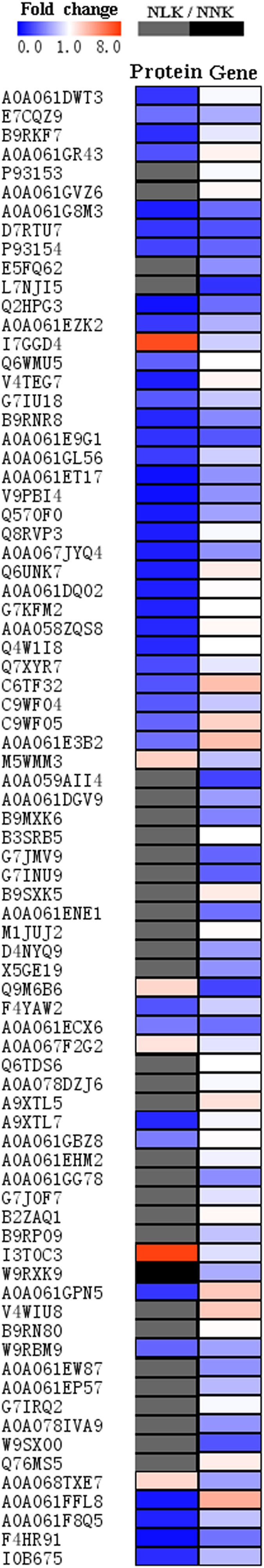
Coordination of protein expression and each corresponding gene expression in K-deficient cotton root and the control.

Compared with the control, expressions of only 4 corresponding genes were significantly up-regulated with FC ≥ 2.0 among 73 down-regulated proteins in the xylem sap (Table 7); there was no corresponding gene with significant down-regulation at FC ≤ 0.5 level. By and large, gene change and protein change had a similar trend, but proteins varied to a greater extent than genes (Fig. 4).

Levels of mRNA are not always consistent with the levels of the corresponding proteins. Three potential reasons accounted for the lack of a strong correlation between mRNA and protein expression levels. First, many complicated and varied post-transcriptional events may result in transcriptome expression levels that might not always be reflected at proteome levels. Second, *in vivo* half-lives of different proteins varied. Finally, there is a significant amount of error and noise in both protein and mRNA experiments.

## Discussion

LK treatment significantly decreased the K content in the xylem sap and different organs, constituting K-deficiency stress. This result was further supported by significant decreases in the leaf area, plant height, root length and surface area, the dry plant weight and protein content in the xylem sap. In the yield, K deficiency of the soil usually lasted, and resulted in inhibition of plant growth. Therefore, we chose a relatively long time of K deficiency (7 days) to explore xylem sap proteins and root genes transcription, and which significantly retarded cotton plant growth as a whole. An analysis of xylem sap proteins showed that 90.31% of the total proteins were secreted proteins, and there were no intracellular protein markers, such as glucose-6-P dehydrogenase and malate dehydrogenase^16^,^24^, indicating that the samples used for protein qualification and quantification were of high quality.

In this study, potassium deficiency inhibited a myriad of protein levels in the xylem sap, with similar inhibition on mRNA levels in the root (Table 7; Fig. 4). In detail, potassium deficiency resulted in the disappearance of not only many peroxidase isoforms but also isoforms of other proteins, such as 1, 3-beta-glucosidase, chitinase and signaling proteins; it also resulted in the appearance of a few proteins in the sampled cotton xylem sap (Table 7; Supplemental Table 1). Many authors have also reported the appearance or disappearance of specific peroxidase isoforms during a particular process or in a particular localization^28^. The diversity of processes that are catalyzed by peroxidases and their numerous genes suggest the possibility of functional specialization of each isoform^29^. Therefore, further research into the function of the proteins that disappeared or appeared in the xylem sap may facilitate an understanding of how cotton responds to potassium deficiency. Additionally, some proteins were identified that have not been previously associated with environmental-stress-related proteins. These proteins were classified into miscellaneous proteins. Research into these proteins and the significant change or absence of uncharacterized proteins between the LK and NK treatments will provide additional information regarding their expression patterns and roles in a plant’s response to environmental stresses and plant root growth.

Proteins in the xylem sap might originate from the secretion of adjacent xylem parenchyma or pericycle cells via the classical (containing a signal peptide) or the non-classical (lacking a signal peptide) secretion pathway^16^,^24^. In our study, 90.31% of the xylem sap proteins were secreted proteins, which was composed of 56.98% classical peptides and 33.33% non-classical peptides; 9.69% were non-secreted proteins, e.g., lactoylglutathione lyase (glyoxalase I) (an enolase). Similarly, enolase has been secreted to the cell wall or extracellular space via immunolocalization even though it lacked a signal peptide^30^. Furthermore, glyoxalase 1 has been identified in the cell wall proteome of the maize primary root elongation zone^31^ and in a cell wall proteomics study of mature alfalfa stems^32^. In addition, the DNA-binding protein histone H_4_ has been unexpectedly found among secreted proteins^25^ and in the cotton xylem sap (Table 7; Supplemental Table 1), although it was predicted as not secreted in the current study (Table 7; Supplemental Table 1).

The non-secreted proteins might result from tracheid development or through direct release after the death of xylem cells, resulting from programmed cell death^33^. The presence of histone in the secretome proteins of the pea root cap is regarded as a global leakage of material from disrupted nuclei in dead cells^25^. Potassium couldn’t increase histone4 gene transcription (Fig. 4), so it might obviously promote histone4 protein translation, cell membrane damage and nuclei disruption, and obviously lead to histone4 increase in xylem sap. On the other side, it might be a positive response due to innate immunity. Moreover, most cell wall proteins belong to multiprotein families, and proteins in the same family can have different cellular localizations. For example, the glycoside hydrolase family proteins in this study were predicted as classical secreted proteins, non-classical secreted proteins, or non-secreted proteins (Supplement Table 1).

Root growth and development are complex processes, comprising cell proliferation, elongation and differentiation, which involve cell wall extension and remodeling by glycosyltransferases (GTs), glycoside hydrolases (GHs) and expansin-like proteins^31^. Changing cell wall properties may affect lateral root emergence from the parent root^34^,^35^. GTs and GHs are two large superfamilies of carbohydrate-active enzymes. All GTs catalyze the transfer of sugar moieties to acceptor molecules. In contrast, GHs hydrolyze bonds that exist between sugar moieties and other molecules. Within each of these superfamilies, nearly all of the proteins were down-regulated or not detectable under the potassium-deficient conditions, suggesting that the cell wall metabolism was largely inhibited by potassium deficiency.

Significantly, potassium deficiency has been shown to inhibit cotton root elongation and lateral root formation^36^, which is consistent with the general decrease in GTs, GHs and expansin-like proteins observed in this study (Table 7; Supplemental Table 1). In addition, fasciclin-like arabinogalactan proteins (FLAs) are necessary for normal Arabidopsis root growth^37^. According to the GO biological process annotation, the xylem cysteine peptidase 1 takes part in developmental programmed cell death and in the regulation of meristem growth, and a peroxidase (A0A061DQ02) takes part in the regulation of meristem growth. These proteins were significantly down-regulated (0.20 fold change (FC) for xylem cysteine peptidase 1; 0.12 FC for A0A061DQ02 and 0.12 FC for FLA 12) or were absent (NLK for FLA 10), further indicating that potassium deficiency inhibited root elongation and branching at the protein level.

**Figure 5:**
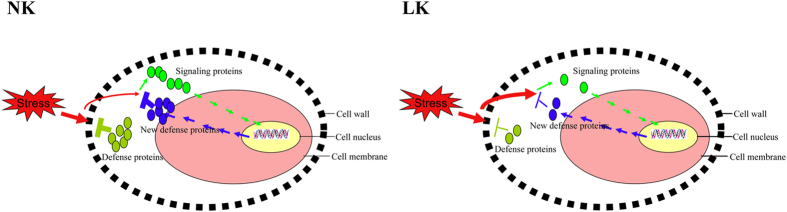
Schematic diagram of comprehensive proteomic-based working model showing how the cellular pathways are being affected under low K (The thicker inhibitory symbol and red arrow denote the stronger function, vice versa. The curved arrow denote that the stress circumvent the defense proteins. The smaller ball (protein symbol) means more proteins. The defense proteins herein include PR-proteins, defense-related cell wall proteins and other defense-related proteins.).

Plants cannot escape environmental harm; thus, adaptability and tolerance to biotic and abiotic stresses is very important for plant survival and growth. The resistance of plants to biotic and abiotic stresses can be divided into pre-formed or passive defense and active defense tactics, responding to stresses that circumvent pre-formed barriers^38^,^39^. Based on significantly different protein levels seen between the LK and NK treatments, it can be concluded the former was related to the cell wall barrier (proline/hydroproline-rich proteins), antifungal proteins and enzymatic inhibitors, whereas the latter was related to recognition and signaling (Fig. 5).

Hydroxyproline-rich glycoproteins (HRGPs) are important plant cell wall components that are involved in plant disease resistant responses, especially in incompatible plant-pathogen interactions, acting as impenetrable physical barriers against pathogen ingress^40^. Arabinogalactan proteins (AGPs) are a class of Hyp-rich glycoproteins, and fasciclin-like AGPs (FLAs) constitute a fourth distinct subclass of AGPs^41^. Lignin is a strengthening polymer that provides not only structural support but also defense against pathogens. Lignin is formed within the plant cell wall matrix by laccase and class Ш peroxidases. Additionally, peroxidase takes part in the production of phytoalexins^42^. Thus, the significant decrease or lack of FLAs or AGPs and peroxidases suggests that potassium deficiency decreased the pre-formed mechanical and biochemical resistance of the cotton root cell wall to pathogen attacks.

One group of proteins that has been closely associated with plant defense is PR proteins. Currently, PR proteins are categorized into 17 families according to their properties and functions, including β-1, 3-glucanases, chitinases, thaumatin-like proteins, peroxidases, non-specific lipid transfer proteins, endo-proteases and protease inhibitors^43^. Chitinases and β-1, 3-glucanases are two important hydrolytic enzymes that are abundant in many plant species. The PR-5 family includes proteins that are related to thaumatin and osmotin, with several members possessing antimicrobial properties^44^. Non-specific lipid-transfer proteins (LTPs) are small, basic, secreted proteins that modulate a plant’s response to biotic stress^45^. Among the proteases inhibitors, polygalacturonase-inhibiting proteins (PGIPs) belong to the large superfamily of Leu-rich repeat (LRR) proteins^46^ and are present in the cell walls of all plants examined to date. These proteins specifically inhibit endo-polygalacturonases (PGs) of fungi but not those of plants or bacteria. The Kunitz trypsin inhibitor exhibits antifungal capabilities and plays an important role in tobacco’s defense response^47^. Peroxidases were also classified as pathogenesis-related proteins involved in plant defense^42^. Generally, PR proteins were significantly down-regulated or lacking under the LK treatment, especially class III peroxidases, indicating a weak passive defense mechanism.

If pathogens circumvent the pre-formed defense system that is weakened in the root apoplast by potassium deficiency, more efficient active defense mechanisms might be required. Active defense requires the plant host to recognize pathogens, signal and activate the related genes to fortify the cell wall, produce phytoalexins, and induce PR proteins.

Given that plant immunity is based on the recognition and constant surveillance of pathogens, the activation of plant defense relies on the recognition of microbial GlcNAc-containing glycans (chitin) that are not inherent to plants themselves; LysM proteins directly or indirectly mediate the recognition of such structures^48^. Therefore, plant LysM proteins are involved in defense signaling against fungal attacks. Some FLAs are likely to be attached to the plasma membrane through a glycosylphosphatidylinositol anchor and may interact with receptor-like kinases, such as wall-associated kinases^49^. Lectins are the only plant proteins that recognize glycoconjugates (glycoproteins, glycolipids, or polysaccharides) on the surfaces of microorganisms, such as bacteria and fungi. The broad spectrum of the carbohydrate-binding specificity of lectins can be interpreted as the successful recognition of different types of sugar-containing receptors by plant cells^50^. Receptor-like protein kinases and NSP-interacting kinase that are localized on the plasma membrane play important roles in biotic stress responses^41^,^51^. Therefore, the significant decrease or lack of these proteins under potassium-deficient conditions might substantially reduce plant recognition, signaling and the subsequent response to environmental stress.

PR proteins and receptor-like kinases are involved in not only biotic but also abiotic stress responses^44^. PR-2 and PR-11 are able to adjust their function according to the nature of the stress, e.g., inhibiting their glucanase and chitinase activities during cold stresses and inducing antifreeze activity. Similarly, thaumatin-like proteins have antifreeze capabilities. The three PR-proteins in the apoplastic space have been shown to inhibit the recrystallization of intercellular ice and prevent the formation of intracellular ice^52^,^53^. Individual gene transformation increases plant resistance to abiotic stress. For example, the overexpression of *LTP3* constitutively enhanced Arabidopsis tolerance to freezing stress^54^; the constitutive expression of a grape aspartic protease gene in transgenic Arabidopsis confers osmotic stress tolerance^55^; and the constitutive expression of a trypsin protease inhibitor confers multiple stress tolerance in transgenic tobacco^56^. Further, a particular subset of AtPrx (a peroxidase) genes and their appreciate expressions are required for the cold tolerance^57^, indicating that a subset of phylogenetically close peroxidases that decrease or are lacking may be essential for potassium-deficiency tolerance (Fig. 2).

Potassium deficiency generally reduces plant resistance to biotic and abiotic stresses^7^,^11^,^12^, due to significantly decreasing or eliminating environmental stress response proteins in cotton seedling xylem sap. Some reports have also shown that potassium deficiency enhances plant resistance to some pathogens^5^,^6^, which is likely due to the significant increase in individual protein isoforms, such as proteinase inhibitors, non-specific lipid-transfer proteins, histones and uncharacterized proteins, under the LK treatment.

It might be possible to enhance plant resistance to environmental stresses using biotechnology to increase the presence of individual genes. However, most plant traits, such as drought and salt tolerance, and insect resistance are controlled by multiple genes. These genes interact via signaling pathways in response to biotic and abiotic stresses^58^. In this respect, improving potassium management can also enhance plant resistance to the environment by positively affecting the activation of many genes, which might be applicable over a broader scope than biotechnological methods.

## Methods

### Cotton seedling culture and potassium deficiency treatment

The cotton cultivar DP 99B was used in this study. The experiments were conducted in a growth chamber under the following conditions: 30/25 °C, 14/10 h light/dark period, and 450 μmol m^−2^ s^−1^ light. The seeds were surface sterilized with 10% H_2_O_2_ for thirty minutes, washed with tap water three times, and soaked for 12 h in tap water. The soaked seeds were germinated and emerged in wet sand. Only those seedlings that emerged well were transferred to a culture solution. The solution contained 2.5 mM KCl, 2.5 mM Ca(NO_3_)_2_, 1 mM MgSO_4_, 0.5 mM NH_4_H_2_PO_4_, 2 mM NaCl, 2 × 10^−4 ^mM CuSO_4_, 1 × 10^−3^ mM ZnSO_4_, 0.1 mM EDTA-FeNa, 2 × 10^−2^ mM H_3_BO_3_, 5 × 10^−6^ mM (NH_4_)_6_Mo_7_O_24_, and 1 × 10^−3^ mM MnSO_4_. This transferring point in time was denoted as 0 d after transferring (DAT). Seedlings at 3 DAT were treated with K at normal K (2.5 mM KCl) and deficient K (0.05 mM) levels. Sodium ions were provided using NaCl to the seedlings that were exposed to low-K conditions; the other mineral nutrients remained unchanged. After 7 d of K treatment, the samples were collected.

### Cotton seedling organ sampling and cation content determination

The seedlings were separated into root, stem including the leaf petiole, and leaf components, oven-dried at 80 °C until a constant dry weight was attained, and weighed. Ions in the dried and ground root, stem and leaf components were extracted with 1 mM HCl for 24 h under room temperature and via rotation (150 rpm). The extracted solution was centrifuged (5000 rpm), and the supernatant liquid was used for ion determination using inductively coupled plasma-optical emission spectrometry (PE-optima 2100 DV, USA)

### Xylem sap collection

At 7 d of potassium deficiency, xylem sap was collected after cutting the stems approximately 5 cm above the junction of the root and the stem under “root pressure”. After thoroughly washing each rootstock surface with distilled water, they were blotted with filter paper, a latex tube was fitted over the rootstock, and the other end of the latex tube was placed into a plastic tube contained in a foam box filled with ice (supplementary Fig. 1). Xylem sap was collected for 48 h and then kept at −80 °C. The xylem sap of each biological replication was collected from 6–8 plants and pooled for protein content determination and from 12–16 plants for label-free protein quantification.

### Protein preparations

Xylem sap was thawed and filtered through 0.2 μm cellulose acetate filters. The filtered xylem sap with the same volume for each biological replication of different K treatments was used to acquire the concentrated proteins using a 3 KD ultra-centrifugal filter device (Amicon Ultra-4, Merk Millipore, Darmstadt, Germany). The concentrated proteins were used for determination and for protein digestion after being precipitated using acetone.

### Determination of antioxidant enzyme activity and their gel activity analysis using modified SDS-PAGE

SOD (EC 1.15.1.1) activity was measured using the NBT photochemical method. One unit of SOD activity was defined as the amount of enzyme required for the 50% inhibition of the rate of NBT reduction at 560 nm, and SOD activity was expressed as unit ml^−1^ xylem sap or unit μg^−1^protein. GPX (EC 1.1.11.7) activity was determined using guaiacol at 470 nm. The 3-mL reaction mixture contained 100 mM potassium phosphate (pH 6.5), 16 mM guaiacol, 10 mM H_2_O_2_ and 3 μL concentrated protein solution. The reaction was initiated upon the addition of concentrated protein solution.

Modified SDS–PAGE was used to separate peroxidase isoforms and SOD isoforms by molecular weight using the prosthetic haem group. The final concentration of SDS was 0.1% (w/v) in all solutions and gels. Samples were diluted in loading buffer to final concentrations of 62.5 mM TRIS-HCl, 0.1% (w/v) SDS, 10% (w/v) glycerol, and 0.002% (w/v) bromophenol blue without reducing compounds and loaded onto the gels without heating. After completion of electrophoresis, for proteins, the gel was stained by coomassie brilliant blue. For SOD activity, the gel was incubated in a solution containing 2.45 mmol/L NBT for 25 min, followed by incubation in 50 mmol/L potassium phosphate buffer (pH 7.8) containing 28 μmol/L riboflavin and 28 mmol/L TEMED under dark conditions. Expression of SOD was achieved by light exposure for 10 to 20 min at room temperature. For GPX activity, the gel was stained with 16 mM guaiacol used as a substrate for peroxidase reaction and 10 mM H_2_O_2_ in 100 Na-acetate buffer pH 5.0.

### Peptide preparations

Protein digestion was performed according to the FASP (filter-aided sample preparation) procedure described by Wiśniewski *et al.*^59^ Briefly, each protein pellet was solubilized in 200 μl SDT buffer (4% SDS, 10 mM DTT, and 150 mM Tris-HCl, pH 8.0) in a boiling water bath for 30 min, amended with DTT to a final concentration of 100 mM, and bathed at 100 °C for 5 min. The solution was then transferred to a 10 kD ultra-centrifugal filter device, amended with 200 μl of UA buffer (8 M Urea, and 150 mM Tris-HCl, pH 8.0), and centrifugally ultra-filtered at 14000 g for 15 min to remove the detergent, DTT and other low-molecular-weight components. Then, the filter device was amended with 100 μl of iodoacetamide (IAA) (50 mM IAA in UA), vibrated (660 rpm, 1 min), placed in darkness at room temperature and centrifuged at 1400 g for 10 min. The tube was amended with 100 μl UA buffer and centrifuged at 1400 g for 10 min, which was repeated twice. The same process was performed with 100 μl 25 mM NH_4_HCO_3_. In the above processes, the liquid filtrate was discarded each time. Finally, the suspended protein was initially digested with 2 μg trypsin (Promega) in 40 μl 25 mM NH_4_HCO_3_ with vibration (600 rpm, 1 min) and subsequently held at 37 °C for 18 h; the resulting peptides were collected as the filtrate.

### UPLC-MS/MS

Peptide mixtures were analyzed by on-line nanoflow liquid chromatography using the EASY-nLC 1000 system (Thermo Finnigan) with a trap column (EASY column SC001 traps; 150 μm × 20 mm (RP-C18)) and analysis column (EASY column SC200; 150 μm × 100 mm (RP-C18)). Each sample was auto-sampled into the trap column and separated by the analysis column at a flow rate of 400 nl/min. The analysis column was balanced with 100% mobile phase A (water solution with 0.1% formic acid and 2% acetonitrile). Peptides were eluted with a linear gradient from 0–45% mobile phase B (water solution with 0.1% formic acid and 84% acetonitrile) for 100 min and 45–100% B for 8 min and maintained at 100% for 12 min. The eluate was electro-sprayed into a Q-Exactive Orbitrap Mass (Thermo Finnigan) for 120 min. The Q-Exactive was operated with one full precursor scan scope (*m/z* 300–1800) (MS^1^ scan) and in a HCD top 10 mode (MS^2^ scan). The resolution was 70,000 for the full scan and 17,500 for the fragments (both specified at an m/z of 200). The exclusion time was 90 sec. Raw files were processed using MaxQuant version 1.3.9.3 (http://www.maxquant.org/) with the iBAQ and match between runs (match time window 2 min) options. For protein identification, the MS/MS spectra were automatically searched by MaxQuant against the target/reverse UniProt Eudicotyledons database (FASTA-formatted protein sequence database). The identified proteins were further statistically and bioinformatically analyzed using Perseus version 1.3.04. The fixed modification was carbamidomethyl (C). The variable modifications were oxidation (M) and acetyl (protein N-term). The initial mass tolerances for the full scans were 6 ppm and 20 ppm for MS/MS. Two missed cleavages were allowed. The peptide and protein false discovery rates (FDR) were both set to 0.01.

### Proteins quantification

The iBAQ (intensity-based absolute quantification) option was used in MaxQuant to calculate the approximate share of each protein in the total proteome based on the sum of the peak intensities^60^. If the iBAQ of a protein was detected two or three times from three biological replicates in the NK and LK treatments, the protein was quantified and compared between the two treatments. If the iBAQ of a protein was detected two or three times from three biological replicates in NK or LK (i.e., only one of the treatments), the protein was termed as being non-detected in LK or non-detected in NK, respectively.

### GO annotations and locations of identified proteins

The gene ontology (GO) annotations in terms of cellular components, molecular functions and biological processes for the identified proteins were obtained from http://www.uniprot.org. The theoretical molecular weights (MWs) and isoelectric points (pIs) of the proteins were collected from http://www.ebi.ac.uk/Tools/seqstats/emboss_pepstats/. The proteins were predicted for secretion with a signal peptide using SignalP (www.cbs.dtu.dk/services/SignalP/) and without a signal peptide using SecretomeP (www.cbs.dtu.dk/services/SecretomeP). In addition, the non-secreted proteins that were predicted by SignalP and SecretomeP were predicted by TargetP (www.cbs.dtu.dk/services/TargetP) for their locations.

### Phylogenetic analysis of peroxidase proteins

A phylogenetic tree was constructed using neighbor joining (NJ) approaches, among which phylogenetic analyses were conducted using MEGA version 5.1 with the following parameters: model (p-distance), bootstrap (1,000 replicates) and gap/missing data (pairwise deletion).

### Calculation of gene expression levels

To obtain comprehensive transcription profile of proteins listed in Table 6 for K-deficient cotton root, we use the Illumina Hiseq2000 to perform high-throughput RNA-seq of K-deficient root and K-efficient root. In total, 8.99 Gb of raw RNA-seq data were generated (BGI-Tech., China).

RNA-seq reads were mapped to the cotton genotype TM-1 genome using Tophat (Version 2.0.8). To measure the gene expression level in LK and NK root tissues, we calculated the expression of each gene using FPKM (Fragments per Kilobase of exon model per Million mapped reads) with Cufflinks (Version2.1.1) (http://cufflinks.cbcb.umd.edu/).

We analyzed the gene (corresponding to proteins listed in Table 6) expression changes of K-deficient cotton root, compared with K-efficient root, and present a heatmap for the coordination of gene transcription and protein expression by using software MultiExperiment Viewer (MeV).

### Statistics

Experiments for effects of potassium deficiency on cotton seeding growth, mineral nutrient contents, xylem sap volume, proteins contents and pH of xylem sap were repeated three times with similar results. Qualification and quantification of proteins in cotton xylem sap of one of three experiments was done. Treatment means were compared using *t*-test.

## Additional Information

**How to cite this article**: Zhang, Z. Y. *et al.* Proteome quantification of cotton xylem sap suggests the mechanisms of potassium-deficiency-induced changes in plant resistance to environmental stresses. *Sci. Rep.* **6**, 21060; doi: 10.1038/srep21060 (2016).

## Supplementary Material

Supplementary Information

## Figures and Tables

**Table 1:** Effects of potassium deficiency on the mineral nutrient contents.

Organs	Treatment	K	Ca	Mg	Fe	Cu	Zn
		mg g^−1^ dry weight					
Root	NK	35.25^**^	30.85	13.42	1.74	0.26	0.82
	LK	20.27	58.75^**^	53.39^**^	3.78^**^	0.57^**^	2.74^**^
Cotyledon	NK	20.37^**^	72.24	23.93	0.73	0.14	0.34
	LK	12.95	100.81^**^	44.44^**^	1.88^**^	0.30^*^	0.85^**^
Forth true leaf	NK	36.69^**^	70.96	28.13	1.89	0.40	0.95
	LK	22.17	104.76^*^	41.61^**^	2.45^*^	0.43	0.99
		mg L^−1^					
Xylem sap	NK	511.31^**^	305.83	50.32	1.61	0.08	1.70
	LK	66.41	429.95^**^	79.35^**^	1.20	0.11	2.65^*^

Emerging cotton seedlings in wet sand were transferred to a normal solution, grown for 3 d, and separated into a K-deficient solution and a new normal solution and grown for 7 d. These cotton seedlings were used for organ and xylem sap sampling and index determination. For each organ, means within each column followed by the * and ** are significantly different according to t-test, respectively, p < 5% and p < 1%, n = 6.

**Table 2 t2:** Effects of potassium deficiency on the physiological characteristics of cotton xylem sap and cotton growth.

Treatment	Xylem sap volume	Protein content	pH	Root	Stem	Leaf
	ml plant^−1^	μg ml^−1^		mg dry weight plant^−1^		
NK	0.48	4.41^**^	6.32^**^	49.6^*^	106.8^**^	196.6^*^
LK	0.91^*^	2.38	5.60	36.0	66.6	165.4

Emerging cotton seedlings in wet sand were transferred into a normal solution, grown for 3 d, and separated into a K-deficient solution and a new normal solution and grown for 7 d. These cotton seedlings were used for organ and xylem sap sampling and index determination. Means within each column followed by the * and ** are significantly different according to t-test, respectively, p < 5% and p < 1%, n = 6.

**Table 3 t3:** The effects of potassium deficiency on activity of GPX and SOD in cotton xylem sap.

Treatment	GPX activity		SOD activity	
	U ml^−1^ xylem sap	U μg^−1^ protein	U ml^−1^ xylem sap	U μg^−1^ protein
NK	130.5**	16.3**	2.6**	0.32**
LK	16.5	6.0	0.2	0.07

Emerging cotton seedlings in wet sand were transferred to a normal solution, grown for 3 d, and separated into a K-deficient solution and a new normal solution and grown for 7 d. These cotton seedlings were used for xylem sap sampling and index determination. Means within each column followed by the ** are significantly different according to t-test, p < 1%, n = 6.

**Table 4:** Qualification and quantification of xylem sap proteins.

Identified proteins	Qualitative peptides	Quantitative peptides in comparison	Non-quantitative peptides in comparison		
			NLK	NNK	Others
Total	258	116	41	5	96
Uncharacterized	72	21	9	4	38

Emerging cotton seedlings in wet sand were transferred into a normal solution, grown for 3 d, and separated into a K-deficient solution and a new normal solution and grown for 7 d. These cotton seedlings were used for xylem sap sampling and three biological replicates of xylem sap samples were used for protein identification, qualification and quantification. More details can be found in Supplementary Tables 1 and 2.

Note: NLK: being non-detectable in low K; NNK: being non-detectable in normal K.

**Table 5:** Classical and non-classical secreted proteins based on the proteins identified in the cotton seedling xylem sap.

Secreted proteins	Qualitative peptides	Quantitative peptides in comparison	Non-quantitative peptides in comparison		
			NLK	NNK	Others
Total	233	109	38	4	82
Classical	147	80	31	1	35
Non-classical	86	29	7	3	47

The identified proteins were predicted as classical secreted proteins using SignalP or non-classical secreted proteins using SecretomeP. More details can be found in Supplementary Tables 1 and 2.

Note: NLK: being non-detectable in low K; NNK: being non-detectable in normal K.

**Table 6:** Effects of K deficiency on the contents of the classified proteins in the cotton xylem sap with ≥ 2- or ≤ 0.5-fold change in comparison between LK and NK and on proteins lacking in either the LK or NK treatment.

Classification	Accession No.	Protein name	Plant species	Theoretical MW/pI	Secrete traits	FC
PR-1	A0A061DWT3	Basic pathogenesis-related protein 1	Theobroma cacao	24.2/4.85	NCSP	0.21^**^
1,3-beta-glucosidase	E7CQZ9	GLU	Gossypium hirsutum	50.3/5.15	CSP	0.44^*^
	B9RKF7	Glucan endo-1,3-beta-glucosidase, putative	Ricinus communis	55.7/6.40	CSP	0.18^**^
	A0A061GR43	O-Glycosyl hydrolases family 17 protein isoform 1	Theobroma cacao	53.1/4.98	NCSP	0.32^**^
	P93153	1,3-beta-glucanase	Gossypium hirsutum	37.6/5.03	CSP	NLK
	A0A061GVZ6	O-Glycosyl hydrolases family 17 protein isoform 1	Theobroma cacao	53.7/8.01		NLK
Chitinase	A0A061G8M3	Acidic endochitinase	Theobroma cacao	36.1/9.87	NCSP	0.11^**^
	D7RTU7	Class I chitinase	Gossypium hirsutum	34.7/6.66	CSP	0.21^***^
	P93154	Chitinase `	Gossypium hirsutum	28.8/6.25	CSP	0.27^***^
	E5FQ62	Class 3 chitinase	Hippophae rhamnoides	31.8/9.05	CSP	NLK
	L7NJI5	Class IV chitinase	Gossypium barbadense	28.6/4.85	CSP	NLK
PR-5	Q2HPG3	Osmotin-like protein I	Gossypium hirsutum	26.5/7.68	CSP	0.07^*^
Protease inhibitor	A0A061EZK2	Kunitz family trypsin and protease inhibitor protein	Theobroma cacao	21.5/6.47	CSP	0.22^*^
	I7GGD4	Proteinase inhibitor	Gossypium arboreum	7.5/4.95	NCSP	7.15^***^
	Q6WMU5	Polygalacturonase-inhibiting protein	Gossypium barbadense	37.1/8.32	CSP	0.38^**^
Proteases	V4TEG7	Carboxypeptidase (serine-type)	Citrus clementina	54.0/4.99	CSP	0.11^**^
	G7IU18	Subtilisin-like serine protease	Medicago truncatula	81.9/7.88	CSP	0.35^**^
	B9RNR8	Aspartic proteinase nepenthesin-2, putative	Ricinus communis	48.8/8.19	CSP	0.15^***^
	A0A061E9G1	Xylem cysteine peptidase 1	Theobroma cacao	39.1/5.66	NCSP	0.20^***^
	A0A061GL56	Cysteine proteinases superfamily protein	Theobroma cacao	39.0/5.40	CSP	0.24^**^
Peroxidases	A0A061ET17	Peroxidase superfamily protein	Theobroma cacao	37.4/6.50	CSP	0.06^***^
	V9PBI4	POD21	Populus tomentosa	37.8/6.66	CSP	0.06^**^
	Q570F0	Peroxidase ATP4a (Fragment)	Arabidopsis thaliana	24.6/4.45	NCSP	0.10^**^
	Q8RVP3	Apoplastic anionic gaiacol peroxidase	Gossypium hirsutum	37.4/4.60	CSP	0.11^***^
	A0A067JYQ4	Peroxidase	Jatropha curcas	36.2/5.22	CSP	0.11^***^
	Q6UNK7	POD9	Gossypium hirsutum	34.9/7.75	CSP	0.11^***^
	A0A061DQ02	Peroxidase superfamily protein	Theobroma cacao	35.6/4.95	CSP	0.12^*^
	G7KFM2	Class III peroxidase	Medicago truncatula	35.8/9.81	CSP	0.13^***^
	A0A058ZQS8	Peroxidase	Eucalyptus grandis	35.5/8.32	CSP	0.15^***^
	Q4W1I8	Basic peroxidase	Zinnia violacea	34.2/8.32	CSP	0.16^***^
	Q7XYR7	Class III peroxidase	Gossypium hirsutum	35.4/9.40	CSP	0.3^**^
	C6TF32	Peroxidase	Glycine max	34.5/9.14	CSP	0.33^***^
	C9WF04	Class III peroxidase	Gossypium hirsutum	35.3/7.68	CSP	0.35^*^
	C9WF05	Class III peroxidase	Gossypium hirsutum	34.0/8.33	CSP	0.40^*^
	A0A061E3B2	Cationic peroxidase 2	Theobroma cacao	39.7/7.47	NCSP	0.44^***^
	M5WMM3	Peroxidase	Prunus persica	35.7/7.29	CSP	2.65^***^
	A0A059AII4	Peroxidase	Eucalyptus grandis	33.9/8.92	CSP	NLK
	A0A061DGV9	Peroxidase superfamily protein	Theobroma cacao	36.8/9.54	CSP	NLK
	B9MXK6	Class III peroxidase	Populus trichocarpa	36.4/9.69	CSP	NLK
	B3SRB5	Putative secretory peroxidase	Catharanthus roseus	35.3/9.10	CSP	NLK
	G7JMV9	Peroxidase family protein	Medicago truncatula	37.6/9.34	CSP	NLK
	G7INU9	Cationic peroxidase	Medicago truncatula	34.7/7.70	CSP	NLK
	B9SXK5	Peroxidase 53, putative	Ricinus communis	35.1/46.3	CSP	NLK
	A0A061ENE1	Peroxidase 24, putative	Theobroma cacao	36.4/9.71	CSP	NLK
	M1JUJ2	Peroxidase 2	Pyrus communis	34.5/9.99	CSP	NLK
	D4NYQ9	Peroxidase	Bruguiera	36.4/10.37	CSP	NLK
	X5GE19	Peroxidase	Carica papaya	37.9/6.73	CSP	NLK
Lipid-transfer protein	Q9M6B6	Non-specific lipid-transfer protein	Gossypium hirsutum	11.9/8.72	CSP	2.43^**^
Oxido-reduction- related proteins	F4YAW2	Copper binding protein 3	Gossypium hirsutum	17.8/4.30	CSP	0.33^*^
	A0A061ECX6	Cupredoxin superfamily protein	Theobroma cacao	22.2/7.97	NCSP	0.48*
	A0A067F2G2	Superoxide dismutase [Cu-Zn]	Citrus sinensis	28.9/7.22	CSP	2.00^**^
	Q6TDS6	Laccase	Gossypium arboreum	63.3/6.73.	CSP	NLK
	A0A078DZJ6	BnaC07g17890D protein (UDP-N-acetylmuramate dehydrogenase activity)	Brassica napus	61.0/7.99	CSP	NLK
Signaling proteins	A9XTL5	Fasciclin-like arabinogalactan protein 10	Gossypium hirsutum	44.3/6.73	CSP	NLK
	A9XTL7	Fasciclin-like arabinogalactan protein 12	Gossypium hirsutum	42.9/5.34	CSP	0.15^***^
	A0A061GBZ8	Receptor-like protein kinase-related family protein	Theobroma cacao	26.7/6.47	CSP	0.49^*^
	A0A061EHM2	NSP-interacting kinase 1	Theobroma cacao	71.6/8.04	CSP	NLK
	A0A061GG78	Cysteine-rich RLK 29 (protein serine/threonine kinase activity)	Theobroma cacao	149.6/5.32	CSP	NLK
	G7J0F7	Lorelei-like-GPI-anchored protein	Medicago truncatula G	18.6/5.61	CSP	NLK
	B2ZAQ1	Peptidoglycan-binding LysM domain-containing related protein	Gossypioides kirkii	47.3/5.30	CSP	NLK
Other stress-related proteins	B9RP09	Heparanase, putative	Ricinus communis	57.3/7.49	NCSP	NLK
	I3T0C3	Histone H4	Medicago truncatula	11.4/12.01		7.54^**^
	W9RXK9	Histone H2A	Morus notabilis	47.3/10.69	NCSP	NNK
Cell wall metabolism	A0A061GPN5	Xyloglucan endotransglucosylase/hydrolase (GH16)	Theobroma cacao	33.3/6.92	CSP	0.21^***^
	V4WIU8	Xyloglucan endotransglucosylase/hydrolase (GH16)	Citrus clementina	31.1/8.68	NCSP	NLK
	B9RN80	Polygalacturonase, putative (GH28)	Ricinus communis	59.0/5.58	CSP	NLK
	W9RBM9	Beta-fructofuranosidase, insoluble isoenzyme CWINV1	Morus notabilis	72.5/5.96	NCSP	0.40*
	A0A061EW87	Alpha-L-arabinofuranosidase 1	Theobroma cacao	75.5/4.70	CSP	NLK
	A0A061EP57	Glycosyl hydrolase superfamily protein isoform 3 (GH1)	Theobroma cacao	66.0/6.60		NLK
	G7IRQ2	Beta-galactosidase (GH35)	Medicago truncatula	91.4/7.58	CSP	NLK
	A0A078IVA9	Beta-galactosidase (GH35)	Brassica napus	114.1/7.38	NCSP	NLK
	W9SX00	Putative beta-D-xylosidase 5	Morus notabilis	87.0/5.98	CSP	NLK
	Q76MS5	LEXYL1 protein(hydrolyzing O-glycosyl)	Solanum lycopersicum	83.1/7.89	CSP	NLK
	A0A068TXE7	Coffea canephora DH200 = 94 genomic scaffold, scaffold_6 (hydrolyzing O-glycosyl compounds)	Coffea canephora	106.4/6.24	NCSP	2.40^**^
Proteins with interacting domains	A0A061FFL8	Curculin-like (Mannose-binding) lectin family protein	Theobroma cacao	51.3/8.17	CSP	0.09^*^
	A0A061F8Q5	D-mannose binding lectin protein with Apple-like carbohydrate-binding domain, putative	Theobroma cacao	49.0/8.27	CSP	0.13^**^
Miscellaneous proteins	F4HR91	Leucine-rich repeat (LRR) family protein	Arabidopsis thaliana	52.7/8.67	CSP	0.05^***^
	I0B675	Epidermis-specific secreted glycoprotein EP1-like protein	Gossypium hirsutum	49.0/6.75	CSP	0.17^**^

Note: LK: low K; NK: normal K; NLK: being non-detectable in LK; NNK: being non-detectable in NK; FC: fold change; CSP: classical secreted proteins; NCSP: non-classical secreted proteins; ^*^P ≤ 0.05; ^**^P ≤ 0.01; ^***^: P ≤ 0.001

**Table 7:** Number of different expression patterns for the different classified proteins in the cotton xylem sap under the LK and NK treatments based on the results presented in Supplemental Table 1.

Classification	SDR	NLK	SUR	NNK	NSC	SUM
PR-1	1	0	0	0	0	1
1,3-beta-glucosidase	3	2	0	0	1	6
Chitinases	3	2	0	0	1	6
PR-5	1	0	1	0	2	4
Protease inhibitor	2	0	1	0	0	3
Proteases	5	0	0	0	1	6
Peroxidases	15	11	2	0	11	39
Lipid transfer protein	0	0	4	0	1	5
Oxido-reductases	3	2	1	0	5	11
Other stress related protein	0	1	2	1	2	6
Signaling	3	5	0	0	5	13
Cell wall metabolism	4	8	1	0	5	18
Proteins with interacting domains	2	0	0	0	1	3
Miscellaneous	3	0	0	0	3	6
Uncharacterized proteins	10	10	3	4	8	35

Note: LK: low K; NK: normal K; NLK: being non-detectable in LK; NNK: being non-detectable in NK; SDR: significant down-regulation; SUR: significant up-regulation; NSC: no significant change.

## References

[b1] Hafsi C., Debez A. & Abdelly C. Potassium deficiency in plants: effects and signaling cascades. Acta Physiol. Plant. 36, 1055–1070 (2014).

[b2] Kumar N., Kavino M. & Kumar A. R. Balanced fertilization for sustainable yield and quality in tropical fruit crops in Balanced fertilization for sustaining crop productivity (eds Benbi D. K., Brar M. S., & Bansal S. K.), 387–405 (International Potash Institute, Horgen, 2006).

[b3] Pettigrew W. T. Potassium influences on yield and quality production for maize, wheat, soybean and cotton. Physiol. Plant. 133, 670–681 (2008). 18331406 10.1111/j.1399-3054.2008.01073.x

[b4] Perrenoud S. Potassium and Plant Health. IPI-Research Topics No3, 365 (International Potash Institute, Basel, 1990).

[b5] Prabhu A. S., Fageria N. K., Huber D. M. & Rodrigues F. A. Potassium and plant disease in Mineral nutrition and plant disease (eds Datnoff L. E., Elmer W. H. & Huber D. M.), 57–78 (The American Phytopathological Society Press, Saint Paul, 2007)

[b6] Amtmann A., Troufflard S. & Armengaud P. The effect of potassium nutrition on pest and disease resistance in plants. Physiol. Plant. 133, 682–691 (2008). 18331404 10.1111/j.1399-3054.2008.01075.x

[b7] Romheld V. & Kirkby E. Research on potassium in agriculture: needs and prospects. Plant Soil 335, 155–180 (2010).

[b8] Härdter R. Crop nutrition and plant health of rice based cropping systems in Asia. Agro-Chemicals News in Brief 20, 29–39 (1997).

[b9] Mondal S. S., Pramanik C. K. & Das J. Effect of nitrogen and potassium on oil yield, nutrient uptake and soil fertility in soybean (*Glycine max*) – sesame (Sesamum indicum) intercropping system. Indian J. Agr.Sci. 71, 44–46 (2001).

[b10] Sweeney D. W., Granade G. V., Eversmeyer M. G. & Whitney D. A. Phosphorus, potassium, chloride, and fungicide effects on wheat yield and leaf rust severity. J. Plant Nutr. 23, 1267–1281 (2000).

[b11] Cakmak I. The role of potassium in alleviating detrimental effects of abiotic stresses in plants. J. Plant Nutr. Soil Sci. 168, 521–530 (2005).

[b12] Oosterhuis D. M., Loka D. A. & Raper T. B. Potassium and stress alleviation: Physiological functions and management of cotton. J. Plant Nutr. Soil Sci. 176 (3): 331–343 (2013).

[b13] Grewal J. S. & Singh S. N. Effect of potassium nutrition on frost damage and yield of potato plants on alluvial soils of the Punjab (India). Plant Soil 57, 105–110 (1980).

[b14] Sharma R. C. & Sud K. C. Potassium management for yield and quality of potato. (2001) Available at: http://ipipotash.org/udocs/Potassium%20Management%20%20Potato.pdf (Accessed: 5^th^ April 2015).

[b15] Hakerlerler H., Oktay M., Eryüce N. & Yagmur B. Effect of potassium sources on the chilling tolerance of some vegetable seedlings grown in hotbeds in Food security in the WANA region, the essential need for balanced fertilization (ed Johnston A. E.) 317–327 (International Potash Institute, Basel, 1997).

[b16] Delaunois B. *et al.* Large-scale proteomic analysis of the grapevine leaf apoplastic fluid reveals mainly stress-related proteins and cell wall modifying enzymes. BMC Plant Biol. 13, 24 (2013). 23391302 10.1186/1471-2229-13-24 PMC3640900

[b17] Chivasa S., Simon W. J., Yu X. L., Yalpani N. & Slabas A. R. Pathogen elicitor-induced changes in the maize extracellular matrix proteome. Proteomics 5, 4894–4904 (2005). 16281185 10.1002/pmic.200500047

[b18] Martinez-Esteso M. J., Sellés-Marchart S., Vera-Urbina J. C., Pedreńo M. A. & Bru-Martinez R. Changes of defense proteins in the extracellular proteome of grapevine (Vitis vinifera cv. Gamay) cell cultures in response to elicitors. J. Proteomics 73, 331–341 (2009). 19822229 10.1016/j.jprot.2009.10.001

[b19] Dahal D., Pich A., Braun H. P. & Wydra K. Analysis of cell wall proteins regulated in stem of susceptible and resistant tomato species after inoculation with Ralstonia solanacearum: a proteomic approach. Plant Mol. Biol. 73, 643–658 (2010). 20496099 10.1007/s11103-010-9646-z PMC3128696

[b20] Szabó E. *et al.* Changes in apoplast protein pattern suggest an early role of cell wall structure remodelling in flagellin-triggered basal immunity. Biol. Plant. 56, 551–559 (2012).

[b21] Dani V., Simon W. J., Duranti M. & Croy R. R. D. Changes in the tobacco leaf apoplast proteome in response to salt stress. Proteomics 5, 737–745 (2005). 15682462 10.1002/pmic.200401119

[b22] Fernandez-garcia N. *et al.* Changes to the proteome and targeted metabolites Of xylem sapin Brassica Oleraceai in response to salt stress. Plant Cell & Environ. 34, 821–836 (2011). 10.1111/j.1365-3040.2011.02285.x 21276013

[b23] Song Y. *et al.* Identification of NaCl stress-responsive apoplastic proteins in rice shoot stems by 2D-DIGE. J. Proteomics 74, 1045–1067 (2011). 21420516 10.1016/j.jprot.2011.03.009 PMC3107904

[b24] Zhang Z. *et al.* Xylem sap in cotton contains proteins that contribute to environmental stress response and cell wall development. Funct. Integr. Genomics 15, 17–26 (2015). 25163431 10.1007/s10142-014-0395-y

[b25] Wen F., Vanetten H. D., Tsaprailis G. & Hawes M. C. Extracellular proteins in pea root tip and border cell exudates. Plant Physiol. 143, 773–783 (2007). 17142479 10.1104/pp.106.091637 PMC1803736

[b26] Driouich A., Follet-Gueye M. L., Vicré-Gibouin M. & Hawes M. Root border cells and secretions as critical elements in plant host defense. Curr. Opin. Plant Biol. 16, 489–495 (2013). 23856080 10.1016/j.pbi.2013.06.010

[b27] Yadav S. K., Singla-Pareek S. L., Ray M., Reddy M. K. & Sopory S. K. Methylglyoxal levels in plants under salinity stress are dependent on glyoxalase I and glutathione. Biochem. Bioph. Res. Co. 337, 61–67 (2005). 10.1016/j.bbrc.2005.08.263 16176800

[b28] Allison S. D. & Schultz J. C. Differential activity of peroxidase isozymes in response to wounding, gypsy moth, and plant hormones in northern red oak (Quercus rubra L.). J. Chem Ecol. 30, 1363–1379 (2004). 15503525 10.1023/b:joec.0000037745.66972.3e

[b29] Cosio C. & Dunand C. Specific functions of individual class III peroxidase genes. J. Exp. Bot. 60, 391–408 (2009). 19088338 10.1093/jxb/ern318

[b30] Edwards S. R., Braley R. & Chaffin W. L. Enolase is present in the cell wall of Saccharomyces cerevisiae. Fems Microbiol. Lett. 177, 211–216 (1999). 10474186 10.1111/j.1574-6968.1999.tb13734.x

[b31] Zhu J. *et al.* Cell wall proteome in the maize primary root elongation zone. I. Extraction and identification of water-soluble and lightly ionically bound proteins. Plant Physiol. 140, 311–325 (2006). 16377746 10.1104/pp.105.070219 PMC1326053

[b32] Watson B. S., Lei Z., Dixon R. A. & Sumner L. W. Proteomics of Medicago sativa cell walls. Phytochem. 65, 1709–1720 (2004). 10.1016/j.phytochem.2004.04.026 15276432

[b33] Alvarez S. *et al.* Characterization of the Maize Xylem Sap Proteome. J. Proteome Res. 5, 963–972 (2006). 16602704 10.1021/pr050471q

[b34] Lewis D. R. *et al.* A Kinetic analysis of the auxin transcriptome reveals cell wall remodeling proteins that modulate lateral root development in arabidopsis. Plant Cell 25, 3329–3346 (2013). 24045021 10.1105/tpc.113.114868 PMC3809535

[b35] Roycewicz P. S. & Malamy J. E. Cell wall properties play an important role in the emergence of lateral root primordia from the parent root. J. Exp. Bot. 65, 2057–2069 (2014). 24619997 10.1093/jxb/eru056 PMC3991740

[b36] Zhang Z. *et al.* Coronatine-induced lateral-root formation in cotton (Gossypium hirsutum) seedlings under potassium-sufficient and -deficient conditions in relation to auxin. J. Plant Nutr. Soil Sci. 172, 435–444 (2009).

[b37] Seifert G. J., Xue H. & Acet T. The Arabidopsis thaliana fasciclin like arabinogalactan protein 4 gene acts synergistically with abscisic acid signalling to control root growth. Ann. Bot. 114, 1125–1133 (2014). 24603604 10.1093/aob/mcu010 PMC4195540

[b38] Dixon R. A. Natural products and plant disease resistance. Nature 411, 843–847 (2001). 11459067 10.1038/35081178

[b39] Nürnberger T. & Lipka V. Non-host resistance in plants: new insights into an old phenomenon. Mol. Plant Pathol. 6, 335–345 (2005). 20565662 10.1111/j.1364-3703.2005.00279.x

[b40] Deepak S. *et al.* Role of hydroxyproline-rich glycoproteins in resistance of pearl millet against downy mildew pathogen Sclerospora graminicola. Planta 226, 323–333 (2007). 17554553 10.1007/s00425-007-0484-4

[b41] Tan L. *et al.* Arabinogalactan-proteins and the research challenges for these enigmatic plant cell surface proteoglycans. Front. Plant Sci. 3, 140 (2012). 22754559 10.3389/fpls.2012.00140 PMC3384089

[b42] Hiraga S., Sazaki K., Ito H., Ohashi Y. & Matsui H. A large family of class III plant peroxidases. Plant Cell Physiol. 42, 462–468 (2001). 11382811 10.1093/pcp/pce061

[b43] Van Loon L. C. & Van Strien E. A. The families of pathogenesis related proteins, their activities, and comparative analysis of PR-1 type proteins. Physiol. Mol. Plant Pathol. 55, 85–97 (1999).

[b44] Rather I. A. *et al.* Molecular cloning and functional characterization of an antifungal PR-5 protein from Ocimum basilicum. Gene 558, 143–151 (2015). 25550044 10.1016/j.gene.2014.12.055

[b45] Sarowar S. *et al.* Overexpression of lipid transfer protein (LTP) genes enhances resistance to plant pathogens and LTP functions in long-distance systemic signaling in tobacco. Plant Cell Rep. 28, 419–427 (2009). 19089429 10.1007/s00299-008-0653-3

[b46] Toubart P. *et al.* Cloning and characterization of the gene encoding the endo polygalacturonase-inhibiting protein (PGIP) of Phaseolus vulgaris L. Plant J. 2, 367–373 (1992). 1303801 10.1046/j.1365-313x.1992.t01-35-00999.x

[b47] Huang H. *et al.* NtKTI1, a Kunitz trypsin inhibitor with antifungal activity from Nicotiana tabacum, plays an important role in tobacco’s defense response. FEBS J. 277, 4076–4088 (2010). 20735473 10.1111/j.1742-4658.2010.07803.x

[b48] Gust A. A., Willmann R., Desaki Y., Grabherr H. M. & Nürnberger T. Plant LysM proteins: modules mediating symbiosis and immunity. Trends Plant Sci. 17, 495–502 (2012). 22578284 10.1016/j.tplants.2012.04.003

[b49] Gens J. S., Fujiki M. & Pickard B. G. Arabinogalactan protein and wall-associated kinase in a plasmalemmal reticulum with specialized vertices. Protoplasma 212, 115–134 (2000). 11543565 10.1007/BF01279353

[b50] Hwang I. S. & Hwang B. K. The pepper mannose-binding lectin gene CaMBL1 is required to regulate cell death and defense responses to microbial pathogens. Plant Physiol. 155, 447–463 (2011). 21205632 10.1104/pp.110.164848 PMC3075774

[b51] Santos A. A., Lopes K. V., Apfata J. A. & Fontes E. P. NSP-interacting kinase, NIK: a transducer of plant defence signalling. J. Exp. Bot. 61, 3839–3845 (2010). 20624762 10.1093/jxb/erq219

[b52] Griffith M. & Yaish M. W. F. Antifreeze proteins in overwintering plants: a tale of two activities. Trends plant sci. 9, 399–405 (2004). 15358271 10.1016/j.tplants.2004.06.007

[b53] Renaut J., Hausman J. F. & Wisniewski M. E. Proteomics and low-temperature studies: bridging the gap between gene expression and metabolism. Physiol. Plant. 126, 97–109 (2006).

[b54] Guo L., Yang H. B., Zhang X. Y. & Yang S. H. Lipid transfer protein 3 as a target of MYB96 mediates freezing and drought stress in Arabidopsis. J. Exp. Bot. 64, 1755–1767 (2013). 23404903 10.1093/jxb/ert040 PMC3617838

[b55] Guo R. *et al.* Constitutive expression of a grape aspartic protease gene in transgenic Arabidopsis confers osmotic stress tolerance. Plant Cell Tiss.Org. 121, 275–287 (2015).

[b56] Srinivasan T., Kumar K. R. R. & Kirti P. B. Constitutive expression of a trypsin protease inhibitor confers multiple stress tolerance in transgenic tobacco. Plant Cell Physiol. 50, 541–553 (2009). 19179349 10.1093/pcp/pcp014

[b57] Kim B. H., Kim S. Y. & Nam K. H. Genes encoding plant-specific class III peroxidases are responsible for increased cold tolerance of the brassinosteroid-insensitive 1 mutant. Mol. Cell 34, 539–548 (2012). 10.1007/s10059-012-0230-z PMC3887832 23180292

[b58] Zhang W. *et al.* Transcriptome sequencing of transgenic poplar (Populus×euramericana’ Guariento’) expressing multiple resistance genes. BMC Genetics 15, (Suppl 1):S7 (2014). 10.1186/1471-2156-15-S1-S7 PMC4118631 25079970

[b59] Wisniewski J. R., Zougman A., Nagaraj N. & Mann M. Universal sample preparation method for proteome analysis. Nat. Methods 6, 359–362 (2009). 19377485 10.1038/nmeth.1322

[b60] Schwanhausser B. *et al.* Global quantification of mammalian gene expression control. Nature 473, 337–342 (2011). 21593866 10.1038/nature10098

