# Supplementary material for: Proteome quantification of cotton xylem sap suggests the mechanisms of potassium-deficiency-induced changes in plant resistance to environmental stresses
Source: Sci Rep. 2016 Feb 16;6:21060. doi: 10.1038/srep21060 (PMC4754703; doi:10.1038/srep21060)
Supplement: Supplementary Information [file srep21060-s1.pdf]

Supplementary Materials to

Proteome quantification of cotton xylem sap suggests the mechanisms of potassium-deficiency-induced changes in plant resistance to environmental stresses

Zhiyong Zhang[1], Maoni Cao[1], Sufang Wang[1], Jingjing Bu[1], Juxiang Tang[1], Fei Li[1], Qinglian Wang[1*], Baohong Zhang[1,2*]

[1] Henan Collaborative Innovation Center of Modern Biological Breeding, School of Life Science and Technology, Henan Institute of Science and Technology, Xinxiang, 453003, China

[2] Department of Biology, East Carolina University, Greenville, NC 27858, US

Supplemental Table 1 List of quantified proteins in comparison of LK and NK or lacking in LK or NK in the cotton xylem sap. Emerging cotton seedlings in the wet sand were transferred into normal solution and grown for 3 d, and then separated into K deficient solution and new normal solution and grown for 7 d. These cotton seedlings were used for xylem sap sampling and identified proteins of quantitative peptides in comparison and lacking in LK or NK were listed as follows.

Classification	accession No.	protein name	plant species	Theoretical Mw/pI	Secrete traits	FC
PR-1	A0A061DWT3	Basic pathogenesis-related protein 1	Theobroma cacao	24.2/4.85	NCSP	0.21**
1,3-beta-glucosidase	E7CQZ9	GLU	Gossypium hirsutum	50.3/5.15	CSP	0.44*
	P93153	1,3-beta-glucanase	Gossypium hirsutum	37.6/5.03	CSP	NLK
	B9RKF7	Glucan endo-1,3-beta-glucosidase, putative	Ricinus communis	55.7/6.40	CSP	0.18**
	A0A061FCI5	Glucan endo-1,3-beta-glucosidase 7-beta-glucanase 7 isoform 3	Theobroma cacao	49.5/5.74	CSP	0.80
	A0A061GR43	O-Glycosyl hydrolases family 17 protein isoform 1 (glucan endo-1,3-beta-D-glucosidase activity)	Theobroma cacao	53.1/4.98	NCSP	0.32**
	A0A061GVZ6	O-Glycosyl hydrolases family 17 protein isoform 1	Theobroma cacao	53.7/8.01		NLK
Chitinase	D7L2X6	PR4-type protein	Arabidopsis lyrata	15.8/8.56	CSP	1.58
	A0A061G8M3	Acidic endochitinase	Theobroma cacao	36.1/9.87	NCSP	0.11**
	D7RTU7	Class I chitinase	Gossypium hirsutum	34.7/6.66	CSP	0.21***
	P93154	Chitinase	Gossypium hirsutum	28.8/6.25	CSP	0.27***
	E5FQ62	Class 3 chitinase	Hippophae rhamnoides	31.8/9.05	CSP	NLK
	L7NJI5	Class IV chitinase	Gossypium barbadense	28.6/4.85	CSP	NLK
PR-5	D2KU75	Thaumatin-like protein	Citrus jambhiri	26.7/7.95	CSP	1.15
	B9NDL0	P21 family protein (thaumatin family)	Populus trichocarpa	24.1/5.73	CSP	1.34**
	Q2HPG3	Osmotin-like protein I	Gossypium hirsutum	26.5/7.68	CSP	0.07*
	A0A061GY18	Osmotin-like protein	Theobroma cacao	27.0/7.73	NCSP	0.45

Protease inhibitor (PR-6)	A0A061EZK2	Kunitz family trypsin and protease inhibitor protein	Theobroma cacao	21.5/6.47	CSP	0.22*
	I7GGD4	Proteinase inhibitor (serine-type)	Gossypium arboreum	7.5/4.95	NCSP	7.15***
	Q6WMU5	Polygalacturonase-inhibiting protein	Gossypium barbadense	37.1/8.32	CSP	0.38**
Proteases	V4TEG7	Carboxypeptidase (serine-type)	Citrus clementina	54.0/4.99	CSP	0.11**
	G7IU18	Subtilisin-like serine protease	Medicago truncatula	81.9/7.88	CSP	0.35**
	B9RNR8	Aspartic proteinase nepenthesin-2, putative	Ricinus communis	48.8/8.19	CSP	0.15***
	A0A061E9G1	Xylem cysteine peptidase 1	Theobroma cacao	39.1/5.66	NCSP	0.20***
	A0A061GL56	Cysteine proteinases superfamily protein	Theobroma cacao	39.0/5.40	CSP	0.24**
	A0A061FR47	Eukaryotic aspartyl protease family protein	Theobroma cacao	46.4/8.96	CSP	0.83
→Peroxidases	A0A061ET17	Peroxidase superfamily protein	Theobroma cacao	37.4/6.50	CSP	0.06***
	V9PBI4	POD21	Populus tomentosa	37.8/6.66	CSP	0.06**
	Q570F0	Peroxidase ATP4a (Fragment)	Arabidopsis thaliana	24.6/4.45	NCSP	0.10**
	Q8RVP3	Apoplastic anionic gaiacol peroxidase	Gossypium hirsutum	37.4/4.60	CSP	0.11***
	A0A067JYQ4	Peroxidase	Jatropha curcas	36.2/5.22	CSP	0.11***
	Q6UNK7	POD9	Gossypium hirsutum	34.9/7.75	CSP	0.11***
	A0A061DQ02	Peroxidase superfamily protein	Theobroma cacao	35.6/4.95	CSP	0.12*
	G7KFM2	Class III peroxidase	Medicago truncatula	35.8/9.81	CSP	0.13***
	A0A058ZQS8	Peroxidase	Eucalyptus grandis	35.5/8.32	CSP	0.15***
	Q4W1I8	Basic peroxidase	Zinnia violacea	34.2/8.32	CSP	0.16***
	A0A061E1J9	Peroxidase N1	Theobroma cacao	35.2/8.33	CSP	0.23
	Q7XYR7	Class III peroxidase	Gossypium hirsutum	35.4/9.40	CSP	0.3**
	C6TF32	Peroxidase	Glycine max	34.5/9.14	CSP	0.33***
	C9WF04	Class III peroxidase	Gossypium hirsutum	35.3/7.68	CSP	0.35*
	Q8RVP4	Bacterial-induced class III peroxidase	Gossypium hirsutum	35.1/7.68	CSP	0.36

C9WF05	Class III peroxidase	Gossypium hirsutum	34.0/8.33	CSP	0.40*
A0A061E3B2	Cationic peroxidase 2	Theobroma cacao	39.7/7.47	NCSP	0.44***
K7ZWQ3	Horseradish peroxidase isoenzyme HRP_1350	Armoracia rusticana	34.3/8.40	CSP	0.68
K4AUE2	Peroxidase	Solanum lycopersicum	35.8/6.35	NCSP	0.87
A0A061GRC6	Peroxidase superfamily protein	Theobroma cacao	35.3/7.36	CSP	1.04
Q8RVP7	Class III peroxidase	Gossypium hirsutum	34.4/8.38	CSP	1.17
P85995	Peroxidase 6 (Fragment)	Vitis vinifera	1.56/6.41		1.19
B9GLK7	Class III peroxidase	Populus trichocarpa	36.6/4.18	CSP	1.25
B5U1R2	Peroxidase 2	Litchi chinensis	37.7/4.47	CSP	1.49*
I3T2D8	Peroxidase	Lotus japonicus	36.3/4.83	CSP	1.52
F6H0Z1	Peroxidase	Vitis vinifera	33.8/7.68	CSP	1.70
G7IM84	Lignin biosynthetic peroxidase	Medicago truncatula	34.1/8.32	CSP	1.72
M5WMM3	Peroxidase	Prunus persica	35.7/7.29	CSP	2.65***
A0A059AII4	Peroxidase	Eucalyptus grandis	33.9/8.92	CSP	NLK
A0A061DGV9	Peroxidase superfamily protein	Theobroma cacao	36.8/9.54	CSP	NLK
B9MXK6	Class III peroxidase	Populus trichocarpa	36.4/9.69	CSP	NLK
B3SRB5	Putative secretory peroxidase	Catharanthus roseus	35.3/9.10	CSP	NLK
G7JMV9	Peroxidase family protein	Medicago truncatula	37.6/9.34	CSP	NLK
G7INU9	Cationic peroxidase	Medicago truncatula	34.7/7.70	CSP	NLK
B9SXK5	Peroxidase 53, putative	Ricinus communis	35.1/46.3	CSP	NLK
A0A061ENE1	Peroxidase 24, putative	Theobroma cacao	36.4/9.71	CSP	NLK
M1JUJ2	Peroxidase 2	Pyrus communis	34.5/9.99	CSP	NLK
D4NYQ9	Peroxidase	Bruguiera	36.4/10.37	CSP	NLK
X5GE19	Peroxidase	Carica papaya	37.9/6.73	CSP	NLK

→Lipid transfer protein	I0IK55	Lipid transfer protein	Eucalyptus pyrocarpa	10.0/8.85	CSP	0.37
	Q9M6B8	Non-specific lipid-transfer protein	Gossypium hirsutum	11.9/8.89	CSP	1.61*
	Q9ZQI8	Non-specific lipid-transfer protein-like protein	Arabidopsis thaliana	16.8/6.98	CSP	1.67*
	D7LZ25	Protease inhibitor/seed storage/lipid transfer protein	Arabidopsis lyrata	12.5/9.02	CSP	1.73*
	Q9M6B6	Non-specific lipid-transfer protein	Gossypium hirsutum	11.9/8.72	CSP	2.43**
Oxido-reductio n related protein	F4YAW2	Copper binding protein 3	Gossypium hirsutum	17.8/4.30	CSP	0.33*
	A0A061ECX6	Cupredoxin superfamily protein	Theobroma cacao	22.2/7.97	NCSP	0.48*
	F4YAW6	Copper binding protein 7	Gossypium hirsutum	19.8/9.17	CSP	0.61
	A3F8V0	Blue copper-like protein	Gossypium hirsutum	15.2/6.06	NCSP	0.89
	F4YAW4	Copper binding protein 5	Gossypium hirsutum	18.0/4.99	CSP	1.05
	B6EBF3	Superoxide dismutase [Cu-Zn]	Gossypium arboreum	15.3/6.50	NCSP	0.77*
	F1CYZ2	Superoxide dismutase [Cu-Zn]	Haberlea rhodopensis	20.5/4.47	CSP	0.90
	A1YQ95	Superoxide dismutase [Cu-Zn] (Fragment)	Cucumis sativus	10.0/5.00	NCSP	1.12
	A0A067F2G2	Superoxide dismutase [Cu-Zn]	Citrus sinensis	28.9/7.22	CSP	2.00**
	Q6TDS6	Laccase	Gossypium arboreum	63.3/6.73.	CSP	NLK
	A0A078DZJ6	BnaC07g17890D protein (UDP-N-acetylmuramate dehydrogenase activity)	Brassica napus	61.0/7.99	CSP	NLK
Signalling	A9XTL5	Fasciclin-like arabinogalactan protein 10	Gossypium hirsutum	44.3/6.73	CSP	NLK
	A9XTL7	Fasciclin-like arabinogalactan protein 12	Gossypium hirsutum	42.9/5.34	CSP	0.15***
	A9XTL8	Fasciclin-like arabinogalactan protein 13	Gossypium hirsutum	43.9/5.55	CSP	0.34
	A9XTK9	Fasciclin-like arabinogalactan protein 4	Gossypium hirsutum	25.8/7.25	CSP	0.84
	A9XTM4	Fasciclin-like arabinogalactan protein 19	Gossypium hirsutum	42.9/6.51	CSP	1.24
	A9XTK6	Arabinogalactan protein 2	Gossypium hirsutum	25.6/9.74	CSP	0.44
	A7J385	Phytocyanin-like arabinogalactan-protein	Gossypium hirsutum	18.8/9.6	CSP	0.56*

	A0A061GBZ8	Receptor-like protein kinase-related family protein	Theobroma cacao	26.7/6.47	CSP	0.49*
	U7DV96	Receptor-like protein kinase	Populus trichocarpa	26.6/7.74	CSP	0.63
	A0A061EHM2	NSP-interacting kinase 1	Theobroma cacao	71.6/8.04	CSP	NLK
	A0A061GG78	Cysteine-rich RLK 29	Theobroma cacao	149.6/5.32	CSP	NLK
	G7J0F7	Lorelei-like-GPI-anchored protein	Medicago truncatula G	18.6/5.61	CSP	NLK
	B2ZAQ1	Peptidoglycan-binding LysM domain-containing related protein	Gossypioides kirkii	47.3/5.30	CSP	NLK
Other stress related proteins	B9RP09	Heparanase, putative	Ricinus communis	57.3/7.49	NCSP	NLK
	M4QNQ1	Chaperonin CPN60-like protein (Fragment)	Mentha piperita	9.2/4.86		0.86
	A0A067G6J3	3-hydroxyisobutyrate dehydrogenase	Citrus sinensis	37.5/6.49	NCSP	0.88
	A0A061GNQ3	Lactoylglutathione lyase	Theobroma cacao	32.5/5.81		1.49*
	I3T0C3	Histone H4	Medicago truncatula	11.4/12.01		7.54**
	W9RXK9	Histone H2A	Morus notabilis	47.3/10.69	NCSP	NHK
Cell wall metabolisms	A0A061G3V1	Expansin-like B1	Theobroma cacao	29.5/4.37	CSP	0.20
	B9SS11	Major pollen allergen Ory s 1, putative (expansin family)	Ricinus communis	27.7/4.34	CSP	0.61
	A0A061DXI3	Expansin-like protein isoform 1	Theobroma cacao	27.9/8.03	CSP	0.64
	A0A061GPN5	Xyloglucan endotransglucosylase/hydrolase (GH16)	Theobroma cacao	33.3/6.92	CSP	0.21***
	F6GXE8	Xyloglucan endotransglucosylase/hydrolase (GH16)	Vitis vinifera	41.7/6.84	NCSP	0.77
	V4WIU8	Xyloglucan endotransglucosylase/hydrolase (GH16)	Citrus clementina	31.1/8.68	NCSP	NLK
	Q84LI7	Polygalacturonase-like protein (GH28)	Fragaria ananassa	51.5/7.68	CSP	0.41
	A0A061E0U0	Polygalacturonase, putative isoform 1 (GH28)	Theobroma cacao	52.7/7.47	NCSP	0.63**
	B9RN80	Polygalacturonase, putative (GH28)	Ricinus communis	59.0/5.58	CSP	NLK
	W9RBM9	Beta-fructofuranosidase, insoluble isoenzyme	Morus notabilis	72.5/5.96	NCSP	0.40*

	A0A061EW87	Alpha-L-arabinofuranosidase 1	Theobroma cacao	75.5/4.70	CSP	NLK
	A0A061EP57	Glycosyl hydrolase superfamily protein isoform 3	Theobroma cacao	66.0/6.60		NLK
	G7IRQ2	Beta-galactosidase (GH35)	Medicago truncatula	91.4/7.58	CSP	NLK
	A0A078IVA9	βeta-galactosidase(GH35)	Brassica napus	114.1/7.38	NCSP	NLK
	W9SX00	Putative beta-D-xylosidase 5	Morus notabilis	87.0/5.98	CSP	NLK
	Q76MS5	LEXYL1 protein(hydrolyzing O-glycosyl)	Solanum lycopersicum	83.1/7.89	CSP	NLK
	A0A068TXE7	Coffea canephora DH200=94 genomic scaffold,	Coffea canephora	106.4/6.24	NCSP	2.40**
	A0A078I819	BnaA03g52860D protein (hydrolase activity,	Brassica napus	95.3/6.71	CSP	0.63**
Proteins with interacting domains	A0A061FX01	Plasmodesmata callose-binding protein 3	Theobroma cacao	25.6/8.26	CSP	0.03
	A0A061FFL8	Curculin-like (Mannose-binding) lectin family protein	Theobroma cacao	51.3/8.17	CSP	0.09*
	A0A061F8Q5	D-mannose binding lectin protein with Apple-like	Theobroma cacao	49.0/8.27	CSP	0.13**
Miscellaneous	I0B675	Epidermis-specific secreted glycoprotein EP1-like protein	Gossypium hirsutum	49.0/6.75	CSP	0.17**
	A0A078DMM5	BnaA10g02880D protein （ATP-binding）	Brassica napus	107.1/7.65	NCSP	0.40
	A0A078DQ53	BnaC04g06650D protein	Brassica napus	19.6/4.80	NCSP	0.70*
	B9IA13	Elongation factor Tu	Populus trichocarpa	49.1/6.91	NCSP	0.71
	A0A068TV45	FCA Coffea canephora DH200=94 genomic scaffold, scaffold_4	Coffea canephora	12.2/8.32	CSP	1.18
	F4HR91	Leucine-rich repeat (LRR) family protein	Arabidopsis thaliana	52.7/8.67	CSP	0.05***
Uncharaterized proteins	V4LA21	Uncharacterized protein	Eutrema salsugineum	46.3/9.30	CSP	0.08***
	A0A059A3Z0	Uncharacterized protein	Eucalyptus grandis	29.5/5.09	CSP	0.08*
	A0A059DA59	Uncharacterized protein (Fragment)	Eucalyptus grandis	28.6/4.55	CSP	0.14***
	A0A022RRL3	Uncharacterized protein	Erythranthe guttata	26.0/9.43	CSP	0.21***
	A0A067FVP8	Uncharacterized protein	Citrus sinensis	35.2/7.99	CSP	0.22**

M5WY13	Uncharacterized protein	Prunus persica	23.4/5.07	NCSP	0.25**
V7D0C4	Uncharacterized protein	Phaseolus vulgaris	32.5/6.00	NCSP	0.27***
V7ANQ0	Uncharacterized protein	Phaseolus vulgaris	81.9/6.69		0.38***
A0A059AD87	Uncharacterized protein	Eucalyptus grandis	46.6/8.63	CSP	0.60*
M1AFQ6	Uncharacterized protein	Solanum tuberosum	15.1/10.63	NCSP	0.61
A5B0T1	Putative uncharacterized protein	Vitis vinifera	192.2/6.99		0.63*
I1JLZ2	Uncharacterized protein	Glycine max	14.5/8.49	NCSP	0.72
R0HV49	Uncharacterized protein	Capsella rubella	19.7/6.77	CSP	0.75
M5XHJ4	Uncharacterized protein (Fragment)	Prunus persica	11.2/8.20	NCSP	0.78
K4CIG7	Uncharacterized protein	Solanum lycopersicum	19.1/8.68	CSP	0.82
A0A022Q547	Uncharacterized protein	Erythranthe guttata	102.5/5.85	NCSP	1.01
K4BUF6	Uncharacterized protein	Solanum lycopersicum	126.3/5.85	NCSP	1.35
M1AE56	Uncharacterized protein	Solanum tuberosum	13.4/4.32	NCSP	1.62
A0A067K243	Uncharacterized protein	Jatropha curcas	27.4/6.47	CSP	1.65**
V4V4R3	Uncharacterized protein	Citrus clementina	106.3/6.38	NCSP	2.11***
V4STA2	Uncharacterized protein	Citrus clementina	82.2/8.57		2.90**
I3SU64	Uncharacterized protein	Lotus japonicus	14.9/4.68	NCSP	NLK
B9RMT3	Putative uncharacterized protein	Ricinus communis	69.1/5.09		NLK
B9TDW3	Putative uncharacterized protein (Fragment)	Ricinus communis	58.3/5.19	NCSP	NLK
C6TFM9	Putative uncharacterized protein (Fragment)	Glycine max	26.8/8.12	NCSP	NLK
D7T2C8	Putative uncharacterized protein	Vitis vinifera	46.4/7.90	NCSP	NLK
V4S7I1	Uncharacterized protein	Citrus clementina	61.3/8.75	CSP	NLK
A0A067JLM8	Uncharacterized protein	Jatropha curcas	54.6/5.88	CSP	NLK
A5ARQ0	Putative uncharacterized protein	Vitis vinifera	47.0/8.27	CSP	NLK

A0A059D710	Uncharacterized protein	Medicago truncatula	25.1/5.71	CSP	NLK
A5BXB5	Putative uncharacterized protein	Vitis vinifera	28.5/8.33	CSP	NLK
A0A022QJT7	Uncharacterized protein	Erythranthe guttata	31.7/7.46	CSP	NHK
S8EN41	Uncharacterized protein	Genlisea aurea	40.7/4.40		NHK
A0A059ATR0	Uncharacterized protein	Eucalyptus grandis	46.7/5.00	NCSP	NHK
S8DWX2	Uncharacterized protein (Fragment)	Genlisea aurea	34.7/9.46	NCSP	NHK

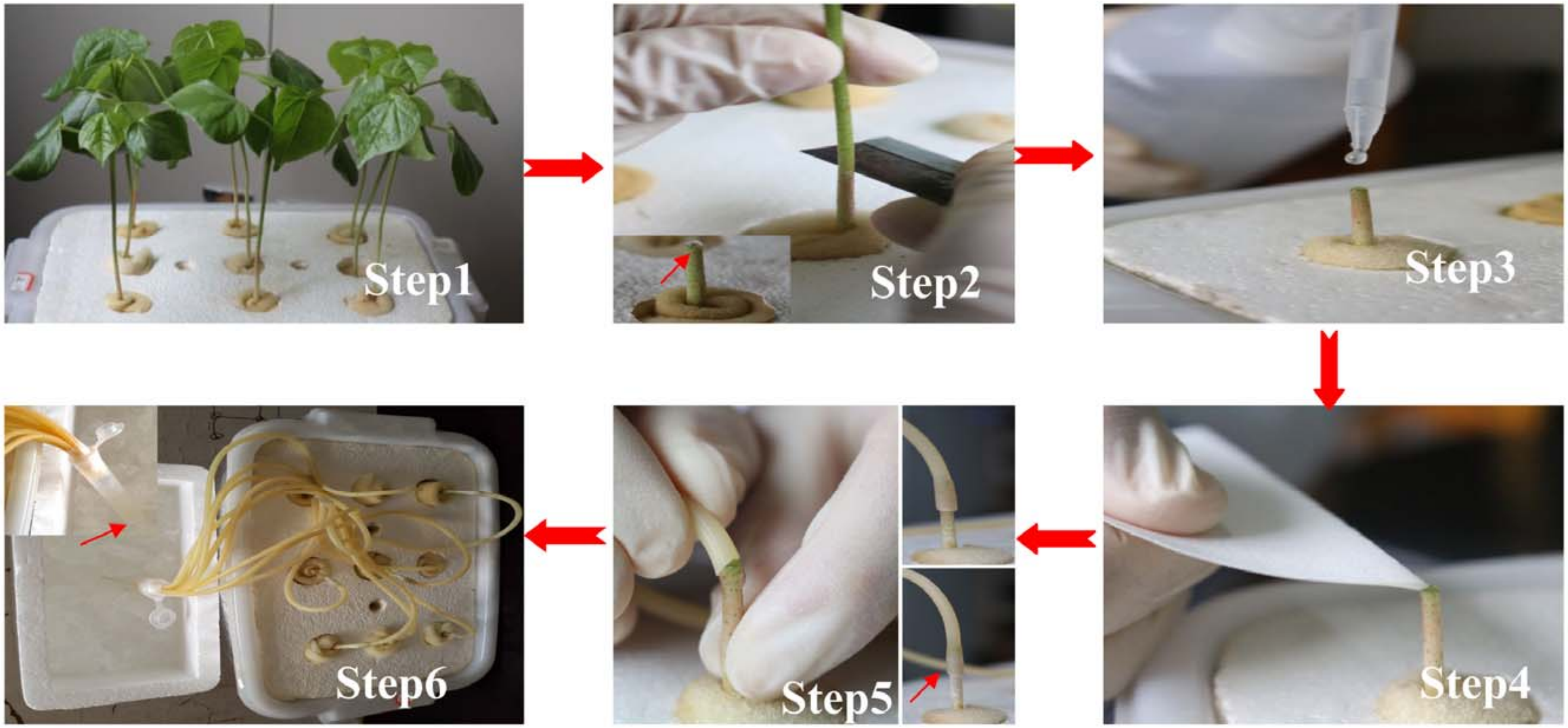

Supplementary Figure 1: Xylem sap collection method (step1: cultured uniform seedlings; step2: horizontally cutting stem and red arrow in this step indicated the first drop of xylem sap secreted immediately after cutting; step3: washing cut surface to remove possible contamination of proteins from broken phloem cell on cutting surface; step4: removing washed water; step5: sheathing the stem and red arrow in this step indicated the Parafilm surrounding the tube and stem; step6: gathering xylem sap and red arrow in this stepindicated the secreted xylem sap.)

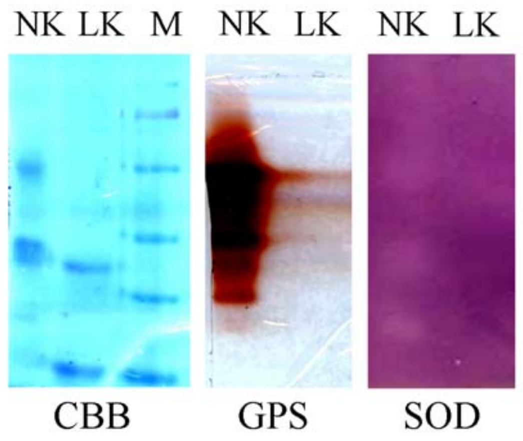

Supplementary Figure 2: Antioxidant enzymes' gel activity analysis (CBB: Coomassie blue staining; GPX: guaiacol-peroxidase; SOD: superoxide dismutase; M: marker; NK: normal K level; LK: low K level)

Supplemental Table S2. Information of *others of non- quantitative peptides in comparison* peptides counted in Table 4 and Table 5. Emerging cotton seedlings in the wet sand were transferred into normal solution and grown for 3 d, and then separated into K deficient solution and new normal solution and grown for 7 d. These cotton seedlings were used for xylem sap sampling and identified proteins including only *others of non- quantitative peptides in comparison* peptides were listed as follows. Information of *quantitative peptides in comparison and NLK and NNK of non-quantitative peptides in comparison* was shown in Supplemental Table1.

Others of non-quantitative peptides in comparison of identified peptides				Secrete traits
Accession No.	Protein name	Plant species	Theoretical Mw(kD)/pI	
K4BNY4	4-hydroxy-4-methyl-2-oxoglutarate aldolase	Solanum lycopersicum	27.78/9.44	
A0A022QKG8	Uncharacterized protein	Erythranthe guttata	49.01/4.81	CSP
A0A022RR84	Uncharacterized protein	Erythranthe guttata	67.23/8.89	NCSP
A0A059ABY3	Uncharacterized protein	Eucalyptus grandis	41.97/9.82	NCSP
A0A059C8Y4	Uncharacterized protein	Eucalyptus grandis	39.94/5.9	NCSP
A0A061E9W7	Plasmodesmata callose-binding protein 3	Theobroma cacao	19.38/6.45	CSP
A0A061F3N5	Glucan endo-1,3-beta-glucosidase	Theobroma cacao	40.88/9.55	NCSP
A0A067GVN3	Uncharacterized protein (Fragment)	Citrus sinensis	75.87/5.69	NCSP
A0A067JRU9	Uncharacterized protein	Jatropha curcas	59.23/9.21	NCSP
I1MKT0	Uncharacterized protein	Glycine max	54.12/8.57	NCSP
A0A067K6I2	Uncharacterized protein	Jatropha curcas	18.56/10.85	NCSP
A0A068TUA6	Coffea canephora DH200=94 genomic scaffold, scaffold_3	Coffea canephora	29.59/7.77	NCSP
A0A068TY33	Coffea canephora DH200=94 genomic scaffold, scaffold_5	Coffea canephora	50.09/7.85	
A0A072U1V6	Beta-amylase-like protein	Medicago truncatula	78.53/5.26	
B7FHS6	Transmembrane protein, putative	Medicago truncatula	30.63/10.35	NCSP
Q95GT7	Maturase K	Nepenthes distillatoria	60.69/9.92	
A0A078EEY5	BnaC04g33590D protein	Brassica napus	10.56/11.18	NCSP
A0A078HI24	BnaC05g30390D protein	Brassica napus	4.75/6.5	NCSP
A0A078HUK8	BnaCnng10570D protein	Brassica napus	6.64/4.03	NCSP
A0A087HFD8	Uncharacterized protein	Arabis alpina	42.76/9.77	NCSP
A0A087HIA4	Uncharacterized protein	Arabis alpina	49.13/7.25	
A0RZC9	Constitutive plastid-lipid associated protein	Solanum lycopersicum	19.78/8.72	NCSP
G7KX54	Non-specific lipid-transfer protein	Medicago truncatula	16.17/9.2	CSP

A5BUS6	Putative uncharacterized protein	Vitis vinifera	164.8/8.14	
A5BZF4	Putative uncharacterized protein	Vitis vinifera	18.15/4.2	NCSP
B9HKA5	Heat Stress Transcription Factor family protein	Populus trichocarpa	40.08/4.9	
B9ILZ3	Histone H4	Populus trichocarpa	10.21/11.75	NCSP
B9R9I9	Peroxidase 66, putative	Ricinus communis	35.8/8.16	CSP
B9RD20	Putative uncharacterized protein	Ricinus communis	21.09/4.55	NCSP
B9SMM0	Peroxidase 27, putative	Ricinus communis	36.06/8.71	CSP
D7KS47	Calcium-dependent protein kinase 30	Arabidopsis lyrata subsp. lyrata	61.49/6.71	
F6GUF3	Putative uncharacterized protein	Vitis vinifera	36.29/4.1	CSP
F8SKC2	Rubber elongation factor	Hevea brasiliensis	14.9/4.79	NCSP
G7IQD7	SET domain protein	Medicago truncatula	73.39/6.32	NCSP
G7JKU3	Gaiacol peroxidase	Medicago truncatula	46.33/9.38	NCSP
G7K809	Uncharacterized protein	Medicago truncatula	21.57/10.11	NCSP
G7KF17	Peroxidase family protein	Medicago truncatula	36.63/8.86	CSP
I1JXW6	Uncharacterized protein	Glycine max	48.87/6.42	CSP
I1KUL3	Uncharacterized protein	Glycine max	23.16/10.1	NCSP
I1MH53	Uncharacterized protein	Glycine max	41.54/9.38	
K4B400	Uncharacterized protein	Solanum lycopersicum	16.45/8.77	NCSP
K4CRR7	Uncharacterized protein	Solanum lycopersicum	46.82/4.36	NCSP
K7K4L8	Uncharacterized protein	Glycine max	28.29/9.76	NCSP
K7LXL8	Uncharacterized protein	Glycine max	201.13/7.08	
M4C9G1	Xyloglucan endotransglucosylase/hydrolase	Brassica rapa subsp. pekinensis	33.12/5.02	CSP
M4D7D6	Uncharacterized protein	Brassica rapa subsp. pekinensis	7.15/4.38	
M5VXG4	Uncharacterized protein	Prunus persica	8.69/8.88	NCSP
M5VXV8	Uncharacterized protein	Prunus persica	23.91/5.36	NCSP
M5WBE2	Uncharacterized protein	Prunus persica	33.26/8.82	NCSP
Q948W7	Zinc-binding protein	Pisum sativum	26.73/6.69	NCSP
Q9SUD0	Multi-copper oxidase type I family protein	Arabidopsis thaliana	62.25/10.05	NCSP
R0GA13	Uncharacterized protein	Capsella rubella	46.18/5.43	
W9REP0	Copper transporter 6	Morus notabilis	16.7/6.9	NCSP
W9S7C8	Aspartic proteinase nepenthesin-2	Morus notabilis	51.73/8.32	CSP
W9S9F5	Transcription factor GTE12	Morus notabilis	41.21/5.23	NCSP
K7MFF6	Uncharacterized protein	Glycine max	53.28/6.52	NCSP
A8J6X3	Cysteine proteinase	Platycodon grandiflorus	39.05/6.84	CSP
A0A022RG42	Uncharacterized protein	Erythranthe guttata	28.23/8.81	CSP
A0A059B7S3	Uncharacterized protein	Eucalyptus grandis	59.75/8.31	NCSP
A0A072UNT2	FAD-binding berberine family protein	Medicago truncatula	59.62/9.27	CSP
A9PDQ1	Plastocyanin-like domain-containing family protein	Populus trichocarpa	23.67/7.04	CSP
A0A061FZ04	Peroxidase superfamily protein	Theobroma cacao	34.71/9.56	CSP

A0A061GQX3	Xyloglucan endotransglucosylase/hydrolase	Theobroma cacao	32.27/8.54	CSP
A0A061GZ24	Pentatricopeptide repeat-containing protein	Theobroma cacao	58.2/6.03	CSP
B9HUZ7	Alpha-galactosidase	Populus trichocarpa	47.03/7.2	CSP
A0A076L4T8	Peroxidase	Cicer arietinum	39.13/7.66	NCSP
A0A078EFF6	BnaC05g22190D protein	Brassica napus	42.14/6.86	NCSP
M1C916	Uncharacterized protein	Solanum tuberosum	62.48/7.17	CSP
A0A078J9R1	BnaCnng42940D protein	Brassica napus	44.44/4.28	NCSP
D7MUR0	Peroxidase 73	Arabidopsis lyrata subsp. lyrata	35.98/10.1	CSP
A9XTK7	Fasciclin-like arabinogalactan protein 2	Gossypium hirsutum	28.31/7.05	CSP
Q9SSZ7	Peroxidase 3	Scutellaria baicalensis	33.9/8.58	CSP
B9RNR9	Aspartic proteinase nepenthesin-2, putative	Ricinus communis	52.32/6.99	NCSP
C9WF09	Class III peroxidase	Gossypium hirsutum	35.73/5.22	NCSP
D7MN89	Pectinesterase	Arabidopsis lyrata subsp. lyrata	63.09/8.88	NCSP
D7MRV5	Beta-xylosidase 4	Arabidopsis lyrata subsp. lyrata	84.33/7.57	CSP
E4MXB5	mRNA, clone: RTFL01-26-I08	Thellungiella halophila	56.38/4.89	CSP
K4ARS5	Uncharacterized protein	Solanum lycopersicum	17.42/9.7	NCSP
K4CYX2	Uncharacterized protein	Solanum lycopersicum	4.23/9.9	NCSP
K7MT09	Uncharacterized protein	Glycine max	35.12/6.32	
K9P1I1	Basic peroxidase swpb7	Ipomoea batatas	35.61/8.95	CSP
A0A087GN76	Uncharacterized protein	Arabis alpina	28.78/8.05	CSP
Q2VAC9	Thaumatin-like protein isoform 2	Ficus pumila var. awkeotsang	24.4/5.84	CSP
Q50LG4	Peroxidase	Nicotiana tabacum	38.53/6.5	CSP
Q8LSM9	Pathogenesis-related protein 5-1	Helianthus annuus	23.95/6.82	CSP
G7K421	Long-chain-alcohol oxidase FAO1-like protein	Medicago truncatula	91.47/7.48	NCSP
Q8W417	Cig3	Nicotiana tabacum	92.89/6.52	NCSP
D7KHX2	Elongation factor 1-alpha	Arabidopsis lyrata subsp. lyrata	105.13/9.56	
F6H6C6	Putative uncharacterized protein	Vitis vinifera	115/5.97	CSP
A0A061GYX9	Peroxidase 68	Theobroma cacao	63.45/9.6	CSP
A0A068V182	Coffea canephora DH200=94 genomic scaffold, scaffold_76	Coffea canephora	50.59/5.21	NCSP
A0A072VDL8	Glucan endo-1,3-beta-glucosidase-like protein	Medicago truncatula	52.04/4.82	CSP
F1BX37	Bacterial-induced peroxidase	Gossypium barbadense	35.76/9.37	CSP
S8DXX8	Uncharacterized protein (Fragment)	Genlisea aurea	25.78/7.57	CSP
M0ZUY3	Uncharacterized protein	Solanum tuberosum	17.92/4.26	NCSP
G7ID83	Plastocyanin-like domain protein	Medicago truncatula	19.76/9.46	CSP

Note: LK: low K; NK: normal K; NLK: being non-detectable in LK; NNK: being non-detectable in NK; CSP: classical secreted proteins; NCSP: non-classical secreted proteins.
